# Telehealth payment parity and outpatient service utilization: evidence from privately insured workers

**DOI:** 10.1093/haschl/qxaf068

**Published:** 2025-04-01

**Authors:** Zhang Zhang, M Kate Bundorf, Qing Gong, Christopher M Shea, Donna Gilleskie, Sean Y Sylvia

**Affiliations:** Department of Health Policy and Management, Gillings School of Global Public Health, The University of North Carolina at Chapel Hill, Chapel Hill 27599, United States; Carolina Population Center, The University of North Carolina at Chapel Hill, Chapel Hill 27516, United States; Department of Health Policy and Management, Bloomberg School of Public Health, Johns Hopkins University, Baltimore 21205, United States; Sanford School of Public Policy, Duke University, Durham 27708, United States; Department of Economics, The University of North Carolina at Chapel Hill, Chapel Hill 27599, United States; Department of Health Policy and Management, Gillings School of Global Public Health, The University of North Carolina at Chapel Hill, Chapel Hill 27599, United States; Carolina Population Center, The University of North Carolina at Chapel Hill, Chapel Hill 27516, United States; Department of Economics, The University of North Carolina at Chapel Hill, Chapel Hill 27599, United States; Department of Health Policy and Management, Gillings School of Global Public Health, The University of North Carolina at Chapel Hill, Chapel Hill 27599, United States; Carolina Population Center, The University of North Carolina at Chapel Hill, Chapel Hill 27516, United States; Sheps Center for Health Services Research, University of North Carolina at Chapel Hill, Chapel Hill 27516, United States

**Keywords:** telehealth payment parity, outpatient service, commercial insurance, worker

## Abstract

Telehealth was catalyzed by the COVID-19 pandemic and has become a new norm in healthcare. In response to the pandemic, some states passed telehealth payment parity legislation, mandating equal payment rates for telehealth and in-person services. We evaluated the relationship between telehealth payment parity and health service utilization, focusing on insured workers in commercial insurance plans. Using the Merative Commercial Claims and Encounters database from 2019 to 2021, we leverage variation in the timing of policy changes across states using a difference-in-difference approach. Payment parity was significantly associated with increased telehealth visits and total outpatient visits but without a notable rise in in-person visits. Furthermore, payment parity was pronounced in increasing telehealth utilization within self-funded large employer plans, while not significantly associated with telehealth visits among fully insured small employer plans. Our findings underscore the important role of payment parity in increasing telehealth service utilization by incentivizing providers. Future policies should support the sustainable integration of telehealth services, shifting from solely focusing on equal payment rates to adopting value-based reimbursement models that improve equitable healthcare access for all employees in commercial insurance.

## Introduction

Telehealth, catalyzed by the COVID-19 pandemic, has become integral to healthcare delivery.^1-4^ After its peak in mid-2020, telehealth usage declined but remains a substantial share of health care, surpassing pre-pandemic levels.^5-7^ In 2023, 74% of physicians surveyed by the American Medical Association reported using telehealth in medical practices, about 3 times the share in 2018.^8^

Federal and state governments as well as private health plans implemented temporary modifications to facilitate telehealth adoption during the pandemic. One key measure in commercial insurance was telehealth payment parity mandates, requiring private insurers to reimburse telehealth and in-person visits equally to incentivize providers to adopt telehealth and ensure that patients maintained access to healthcare services.^9^ In the absence of payment parity mandates, private health insurance typically reimburses telehealth services at lower rates than in-person services.^9^ Before the pandemic, 15 states had implemented payment parity, while with the onset of the pandemic in March 2020, an additional 10 states implemented temporary mandates with 9 (excluding Texas) extending this mandate through 2021.^9^ Evidence on the impact of payment parity is mixed. Some studies showed that state-level telehealth coverage requirements had no significant association with telehealth adoption among underserved populations and telehealth usage in emergency departments prior to the pandemic.^10,11^ Recent studies revealed that payment parity was associated with higher telehealth usage at community health centers, higher diagnosis rates of cancer, and increased contraceptive visits via telehealth during the pandemic.^12-14^ Payment parity has also motivated hospitals to expand telehealth services to improve access to critical services.^15^

At both state and federal levels, there is ongoing debate about permanently adopting payment parity.^15-19^ On the one hand, there is concern that inadequate compensation without parity may disincentivize providers, limiting patient options.^10,12,20^ On the other hand, there is a debate regarding whether the costs of telehealth services might be lower than those of in-person visits.^21^ The incremental services patients use in response to higher rates for telehealth services could represent low-value care.^22^ These concerns stem from the possibility that easier access to healthcare via telehealth could lead to excessive use of healthcare services, potentially overloading the health system and causing inefficiencies.^23^ Also, parity laws may encourage clinicians to abandon their physical practices.^23^ Although the effectiveness of payment parity policies has been explored in specific contexts, there is limited research on their broader impact on outpatient service utilization across diverse populations during and beyond the onset of the pandemic (March-April 2020).^9,24,25^ Additionally, state-level payment parity primarily applies to fully insured health plans offered by state-licensed issuers, constituting most small employer group plans.^26^ In contrast, self-funded plans, common among large employers, are exempt due to the Employee Retirement Income Security Act of 1974 (ERISA) and are regulated at the federal level instead.^26^

Therefore, this study focuses on understanding the role of payment parity in outpatient service utilization among insured workers. Using the 2019-2021 Merative Commercial Claims and Encounters database (Merative data), we leverage exogenous regulatory changes for a difference-in-differences (DiD) design to compare outpatient visits in states with and without payment parity mandates. Our hypothesis was that payment parity increased the use of telehealth visits by enhancing providers’ incentives. The effect on the total number of outpatient visits, however, depends on the extent to which telehealth serves as a substitute for in-person visits. Thus, we examine the effects of payment parity on the number of telehealth and in-person visits, and total outpatient visits. We also examined whether the effects of payment parity varied between workers enrolled in self-funded (self-insured by large firms) health plans and those with fully insured (employer contracted with insurance companies) health plans.

## Methods

### Data and study sample

Our data source was the Merative Commercial Claims and Encounters database (previously named IBM MarketScan data), a nationwide sample of individuals enrolled in privately insured employer-based insurance and Medicare Advantage plans in the United States.^27^ The Merative data include records of patient enrollment, inpatient and outpatient medical claims, outpatient prescription drug claims, and lab tests.^27^ In this study, we focused on the period from 2019 to 2021. We used the data from outpatient medical claims, which include doctor's offices, hospital outpatient facilities, emergency departments, or other outpatient facilities. In addition, we acquired the publicly available COVID data tracker from the Centers for Disease Control and Prevention (CDC) to control for differences across states measured by COVID-19 cases per 1 000 000 population.^28^ A sample selection flowchart is included in Figure S2.1.

### Measures

Our analyses focus on provider visits subject to payment parity policies (ie, nonhospitalization). The scope of services in our study included outpatient services such as office visits, telephone, and video visits. We used telehealth-specific codes: place of service code (02), modifier codes (GT, GQ, 95, GO), or telehealth-specific CPT codes to identify the specific service types (Tables S2.1 and S2.2). We describe our primary outcome of interests, *Telehealth service utilization*, using two variables:

1. An indicator of any telehealth visits.
2. Number of telehealth visits.

We also measured the number of in-person visits and the total number of outpatient visits (which is the sum of telehealth and in-person visits). Our dependent variables are measured at the individual-month level.

### Statistical analyses

We leveraged differential policy adoption by states to identify the causal effects of interest using a two-way fixed-effects DiD analysis. We drew on literature documenting state-level telehealth payment parity changes in private insurance payment parity in response to the pandemic (Figure S2.2).^9^ To analyze the impact of telehealth payment parity mandates, we created a binary variable to indicate the treated and control observations. The *treated* group included individuals in the 9 states that moved to payment parity in response to the pandemic onset in March 2020 and continuously implemented the policy until the end of 2021 (Figure S2.3).^12^ The *control* group comprised those individuals in the states that had not implemented payment parity by 2021, either prior to or during the pandemic (Figure S2.3).^12^ In our main analysis, we excluded individuals in the 15 states that exercised payment parity both before and after the onset of the pandemic throughout the 3-year period (*always* group) (Figure S2.3).^12^ We compared trends, among the control and treated groups, in the outcomes of interest by month from 2019 to 2021. We estimated the pre- and post-difference (where pre- and post-reference the onset of the pandemic) between the 2 groups, adjusting for individuals’ age, sex, occupation, rurality, comorbidity, and COVID-19 cases in that state. Rurality was measured based on whether the geography code belongs to a Metropolitan Statistical Area (MSA), indicating urban areas, or a non-MSA, indicating rural areas. Comorbidity was measured by the Elixhauser Comorbidity Index.^29^

In the heterogeneity analysis, we measured the health plan types by an indicator variable (0 = self-funded health plans; 1 = full insured health plans). We investigated how payment parity was differentially associated with the utilization across health insurance types of self-funded and fully insured plans.

We conducted additional analyses to assess the robustness of our study design and results. First, the validity of our DiD design relies on the assumption of parallel trends. We assessed this assumption using an event study regression, interacting the treatment indicator and the indicator for each year-month in our study period, to infer whether trends were parallel in the treated and control groups prior to policy adoption. Second, we conducted different model specifications to estimate the effect of the policy by multiple extensions (Tables S2.7-S2.11). Analyses were performed using Stata, version 17.1 (StataCorp LLC), and Python. The study utilized only deidentified insurance claim data, was classified as not involving human participants, and thus was exempt from informed consent requirements by the institutional review board.

### Limitations

This study has several limitations. First, the Merative database lacks information on provider characteristics, such as hospital size and technological readiness, which may influence telehealth adoption.^30-32^ Second, the dataset, though national in scope, is not geographically representative, limiting the generalizability of findings. For example, the dataset may oversample individuals from higher socioeconomic status groups with insurance. This skew can lead to an overestimation of telehealth adoption rates, as these groups are more likely to have access to necessary technologies and broadband connectivity.^33,34^ The data primarily includes workers in commercial insurance, excluding insights into underserved populations, such as those with Medicaid or the uninsured. The absence of demographic information, including race and ethnicity, also constrains the analysis of disparities in telehealth access.^34^ In our future study, we will evaluate the effects of the telehealth expansion regulations on health service utilization among underserved and marginalized populations across diverse demographic and socioeconomic groups. Third, while we control for state-level COVID-19 incidence in our analysis, we acknowledge that our estimation model does not capture all aspects of the pandemic's impact. The regulations of lockdowns and social distancing measures in different states could have influenced patient behavior and provider availability.^35,36^

## Results

### Characteristics of study population

The study sample for the main analysis included 1 051 344 person-month utilization observations among 29 204 insured working-age adults (Table S2.3). During the pre-period, the demographic characteristics of workers, including age and sex, were similar between the treated and control groups. There existed some differences in socioeconomic characteristics. For example, self-funded plans, as opposed to fully insured health plans, are more prevalent in the treated group than the control group. A higher proportion of individuals reside in rural areas in the control group than the treated group. Chronic health condition prevalence is similar across groups, with a slightly higher proportion of comorbidity in the control group.

### Descriptive analysis

**Figure 1.**
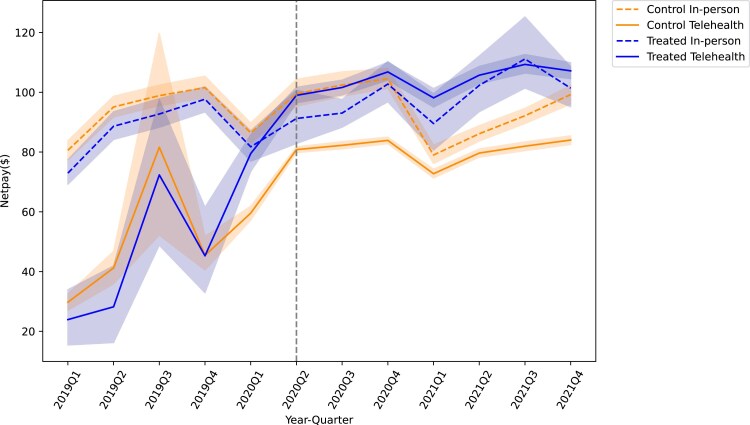
The net payment across service types by person by quarter, 2019-2021. Authors’ analysis of data from the Merative Commercial Claims and Encounters database, January 2019-December 2021. The solid line represents telehealth visits, and the dash line represents in-person visits. (We acknowledged that the sample size for telehealth visits was small, so some noise existed during the pre-period).

**Figure 2.**
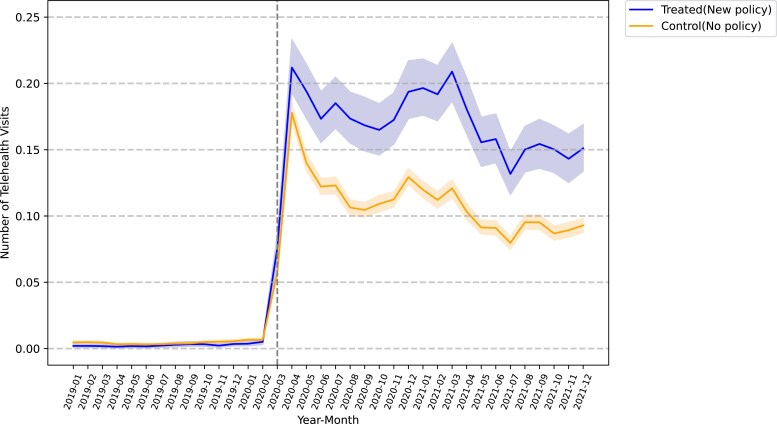
Number of telehealth visits by month, 2019-2021. Authors’ analysis of data from the Merative Commercial Claims and Encounters database, January 2019-December 2021. The analysis is based on the full sample of 29 204 insured working-age adults with 1 051 344 person-month utilization observations.

First, we plotted the trend of net payment by quarter across service types for telehealth and in-person services in treated and control groups (Figure 1). Before March 2020, payments for telehealth services were consistently lower than in-person visits across both groups. Following the implementation of payment parity policies, telehealth payments in the treated group approached parity with in-person payments, suggesting that the mandate effectively influenced telehealth payment rates. Second, we plotted the trend of telehealth visits in treated and control states from 2019 to 2021 (Figure 2). Prior to March 2020, the number of telehealth visits was extremely low. A sharp increase in the number of telehealth visits existed in both the treated and control groups after the onset of the pandemic. However, after implementing payment parity, the treated group had more telehealth visits than the control group, on average, during the onset of the pandemic in 2020 to the end of 2021.

We differentiated the analyses by insurance plan type because state-level payment parity regulation typically applies to fully insured plans rather than self-funded plans. Self-funded large employers are generally not subject to state law but are regulated at the federal level. We plotted the trends in telehealth visits among the treated and control groups for workers insured by self-funded plans and those insured by fully insured contracted group plans (Figure S2.4). Interestingly, the difference in telehealth visits between the treated and control groups was more pronounced among those with self-funded plans than those in fully insured plans, which was contrary to our expectations.

### Difference-in-differences estimations

Table 1 presents the DiD regression results, which measured the impact of payment parity on health service utilization by comparing the treated and the control groups during the posttreatment periods relative to the pretreatment period while controlling for other relevant factors. Table 1 includes the interaction (treatment effect) coefficient for several outcomes. Compared with the control group, telehealth payment parity was associated with a 2.4% point increase in the probability of having at least 1 telehealth visit (*P* < 0.1) (relative to the pre-period mean of 0.7% points or post-period mean of 7.6% points) (Table 1, Panel A, Column 1) and 0.064 more telehealth visits by month on average in the treated group (*P* < 0.05) (relative to the pre-period mean of 0.008 or post-period mean of 0.110) (Table 1, Panel A, Column 2). These results indicate that during and following the onset of the pandemic, telehealth payment parity effectively increased telehealth utilization and the increase persisted through the end of 2021.

**Table 1.** Regression results: impact of telehealth payment parity on outpatient service utilization, 2019-2021.

	Telehealth		In-person	Total
	(1)	(2)	(3)	(4)
	Probability of any visits	Number of visits	Number of visits	Number of visits
	b/se/ci95	b/se/ci95	b/se/ci95	b/se/ci95
Panel A				
Full sample (*N* = 391 608)				
Treated × Post-period	0.024*	0.064**	−0.034	0.029*
	0.013	0.030	0.025	0.015
	−0.001,0.049	0.004,0.124	−0.085,0.016	−0.002,0.060
Pre-period mean	0.007	0.008	0.902	0.910
Post-period mean	0.076	0.110	0.908	1.018
Panel B				
Self-funded plans (*N* = 337 932)				
Treated × Post-period	0.026*	0.070**	−0.030	0.041***
	0.013	0.032	0.029	0.015
	−0.001,0.052	0.006,0.135	−0.088,0.029	0.010,0.071
Pre-period mean	0.006	0.008	0.908	0.916
Post-period mean	0.077	0.111	0.907	1.018
Panel C				
Fully insured plans (*N* = 53 676)				
Treated × Post-period	0.008	0.009	−0.054	−0.045
	0.010	0.019	0.035	0.040
	−0.012,0.027	−0.029,0.047	−0.124,0.015	−0.125,0.035
Pre-period mean	0.007	0.008	0.870	0.878
Post-period mean	0.069	0.101	0.915	1.016

Authors’ analysis of data from the Merative Commercial Claims and Encounters database, 2019-2021.

Selected coefficients from a difference-in-differences estimation model where the treated group is individuals residing in states that implemented telehealth payment parity at the onset of the pandemic, and those residing in states never implementing parity are the control group. Controls include age, sex, occupation (payroll type and type of work), rurality, comorbidity index, and state-level COVID-19 cases. Rurality is measured based on whether the geography code belongs to a Metropolitan Statistical Area (MSA), indicating urban areas, or a non-MSA, indicating rural areas. The last row of each of the 3 panels reports the mean of the dependent variable for the control group states in the pre-period. The full models are included in Tables S2.4-S2.6.

^*^ *P* < 0.10, **P < 0.05, ***P < 0.01. Standard errors in parentheses 95% confidence intervals in brackets.

In contrast, telehealth payment parity was associated with a slight reduction in in-person visits in the treated group by 0.034 visits, but the difference was not statistically significant (Table 1, Panel A, Column 3; Figure S2.5). On net, payment parity was associated with a significant increase in the total number of visits, with an average of 0.029 more visits in the treated states (*P* < 0.1) (Table 1, Panel A, Column 4; Figures S2.6). These findings suggest that telehealth payment parity resulted in the partial substitution of some in-person visits with telehealth visits. However, in-person and telehealth services are not perfectly substituted or completely interchangeable. Telehealth payment parity was associated with an increase in overall outpatient utilization, including telehealth visits.

### Heterogeneous effects

To understand the heterogeneous impact of the treatment effect across health plan types, we ran the same regression on the samples separately by plan type: self-funded plans (second panel) and fully insured health plans (third panel) in Table 1. For workers with self-funded plans, there was an average increase of 0.070 telehealth visits resulting from payment parity (*P* < 0.05) (Table 1, Panel B; Table S2.5). Additionally, payment parity was significantly associated with an increase of 0.041 visits in the overall number of visits (*P* < 0.05) (Table 1, Panel B; Table S2.5). However, for workers with fully insured plans, there was no statistically significant difference in telehealth visits, in-person or total visits (Table 1, Panel C; Table S2.6). While state-level insurance mandates primarily target fully insured health plans, telehealth payment parity mandates had a more pronounced effect among workers in self-funded health plans than in fully insured health plans.

### Robustness check

The results from the event study provided support for the parallel trends assumption underlying the DiD design (Figure S2.7). There was a lack of pre-trend difference in outcomes of interests. Consistent with the DiD regression results in Table S2.3, telehealth utilization experienced a significant increase after the onset of the COVID-19 pandemic. We estimated multiple extensions to assess the robustness of estimates to different model specifications and sample choices. To control the confounders, we added demographic characteristics, socioeconomic factors, health conditions, state-level COVID-19 cases, and region (Tables S2.7-S2.10). All models include state-fixed effects and month-fixed effects. Results appear highly stable with or without controlling for these factors. As yet another robustness check, we redefined the control group to include the “always group” that had implemented the payment parity before the onset of the pandemic (Table S2.11). We also compared the treated group and “always group” (Table S2.12). Overall, these results align with our main findings (for additional details on estimation, refer to Tables S2.7-S2.12).

## Discussion

Our study used the Merative data 2019-2021 to investigate the relationship between payment parity and telehealth utilization among insured workers with commercial insurance. We found that the telehealth payment policy, driven by supply-side incentives, effectively increased the telehealth service payment rate for providers (Figure 1) and, thus, enhanced telehealth utilization (Figure 2). However, the mandate may exacerbate disparities in telehealth access between employees with self-funded health plans (typically offered by large firms) and those with fully insured health plans (typically offered by smaller firms).

Our primary findings suggest that the payment parity plays a significant role in enhancing financial incentives and promoting the utilization of telehealth services. In treated states, the use of telehealth services surged more significantly than in control states both during and after the onset of the pandemic. This trend indicated the responsiveness to policy incentives and increased willingness to adopt telehealth services. We recognize that other telehealth expansion-related policies may have been implemented at the same time as the payment parity. However, other concurrent policies, like telehealth coverage parity mandates (requiring insurers to cover telehealth services if in-person equivalents are covered but not at the same rate), had less variation across states, with 40 states and D.C. adopting them by 2021. Including coverage parity mandates did not confound the evaluation of payment parity effects. Additionally, pandemic mitigation measures (eg, mask mandates and stay-at-home orders) were potential confounders. However, excluding the onset and peak pandemic period in 2020 showed similar trends between treated and control groups through 2021.

The relationship between payment parity in telehealth and in-person services is crucial for policy considerations. While telehealth utilization surged, in-person visits experienced a modest, statistically insignificant decline. This suggests that telehealth partially substitutes for in-person care but also expands overall outpatient service utilization, particularly among self-funded health plans. Policymakers must balance concerns regarding potential overutilization while ensuring that expanded access to telehealth translates into improved patient health outcomes rather than unnecessary expenditures. These findings are relevant not only to commercial insurance but also inform Medicare and Medicaid decisions in evaluating and extending telehealth waivers.

Furthermore, we found that the impact of payment parity on telehealth utilization is heterogeneous by health plan type. Although state-level parity laws primarily regulate small health plans, the mere indirect policy effect via spill-over is still more pronounced among employers with self-funded plans, typically large employers. Such spillovers may occur as providers streamline their services across all patient categories to maintain consistency and efficiency. The regulation may also generate positive market signals to encourage fixed-cost investment in the health system infrastructure, such as the development of health information systems and telehealth technologies. These system-level changes may generate spill-over effects for enrollees with self-funded plans. Additionally, compared with fully insured health plans, self-funded large employer plans have greater flexibility in selecting and designing health plans for their employees and, if contracting, have greater bargaining power to negotiate a more favorable payment rate with insurance companies.^37^ Self-funded plans are also better to embed digital health within an integrated care approach. For example, large employers often incorporate digital health programs, such as wellness, telehealth, and wearable health technologies into their health plans, which have been proven to improve employee health outcomes and substantially drive down employer and employee premiums over time.^38^ However, the most innovative digital health programs and technologies are typically absent from the small group market.^38^ In 2020, about 11.9 million individuals were enrolled in small group plans, and 41.5 million were enrolled in self-funded plans provided by large employers.^39^ In the case of telehealth, payment parity laws may have created incentives for providers to offer telehealth, but people with more generous coverage may have been more likely to use these services. Looking ahead, beyond the providers’ incentives created by payment parity, it is important to develop a financial structure that makes telehealth accessible and affordable for all, especially those in small firms with fully insured health plans.

Our study was an early investigation of the issue based on outpatient service utilization from 2019 to 2021. Going forward, it will be important to continue to monitor the trend between the states with and without telehealth payment parity. In addition, our future research aims to determine whether telehealth payment parity not only increases access to care but also improves the quality of that care, measured both by subsequent utilization (eg, inpatient service and Emergency Department service) as well as health outcomes. It will be important to ensure that reimbursement policies effectively position telehealth as a substitute for more expensive and less accessible care without leading to unnecessary expenditures or increased use of in-person services. While payment parity may have served as a temporary catalyst for telehealth adoption during the pandemic, its long-term sustainability requires alignment with more sustainable principles. With the expiration of the public health emergency, the post-pandemic era offers a unique opportunity to reshape telehealth policies. We advocate for a shift from solely focusing on payment parity to adopting value-based reimbursement models. For example, tiered reimbursement rates—higher for evidence-based telehealth use that replaces costlier in-person care or improves outcomes—could prevent unnecessary expenditures while preserving access. With healthcare evolving in the face of technological advancements and changing patient needs, policies must adapt to ensure that telehealth service is not only accessible but also effective, equitable, value-added, and financially sustainable.

## Conclusion and policy implications

Our findings underscore the importance of telehealth payment parity in increasing outpatient service utilization by incentivizing provider adoption and improving access to care. However, future policies must address two critical challenges highlighted by our findings. First, while telehealth partially substitutes in-person care, it also expands overall outpatient utilization, necessitating measures to prevent the potential overuse of low-value services. Second, differences in telehealth utilization by health insurance groups persist, as self-funded plans benefit more than fully insured plans due to the spill-over effects. Such discrepancies across types of plans reflect the more fundamental issues in the healthcare system. Targeted policies are needed to ensure equitable access across all employee groups from telehealth expansion. Policymakers should refine payment parity mandates to enhance access and support sustainable integration of telehealth, transitioning from a focus solely on equal payment rates to value-based reimbursement models. These findings are not only relevant for private insurance but also provide important evidence for Medicare and Medicaid in designing the payment model for telehealth services.

## Supplementary Material

qxaf068_Supplementary_Data

## Data Availability

The data in this analysis were shared through a data use agreement with Merative. Copyright@2022 Merative. All Rights Reserved. Per the data use agreement, that data is not permitted to be shared.
