# Supplementary material for: Telehealth payment parity and outpatient service utilization: evidence from privately insured workers
Source: Health Aff Sch. 2025 Apr 1;3(4):qxaf068. doi: 10.1093/haschl/qxaf068 (PMC12013821; doi:10.1093/haschl/qxaf068)
Supplement: qxaf068_Supplementary_Data [file qxaf068_supplementary_data.zip › 2.Supplement.docx]

##### **Appendix**

##### **Supplemental Figure S2.1. Sample Selection Flow Chart**

**
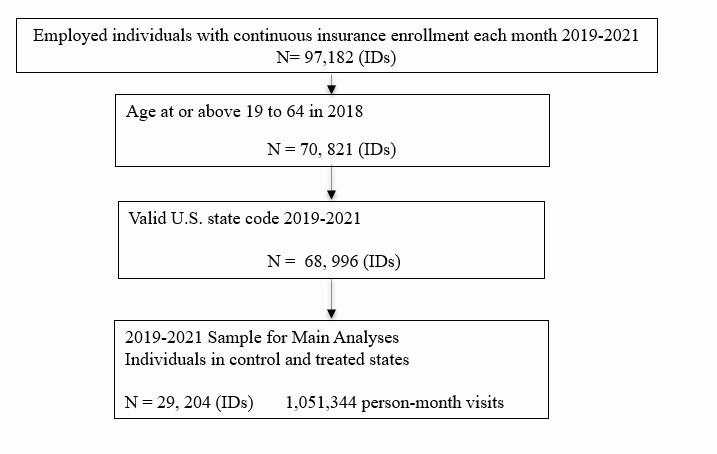
**

**Note:** To avoid confounding concerns related to changes in employment or insurance that coincide with payment parity changes or health changes, we focus on employed individuals aged 19 to 64 who were continuously enrolled in commercial insurance from 2019 to 2021. Using the definition from the Centers for Medicare & Medicaid Services (CMS) Chronic Conditions Warehouse, we created the Elixhauser Comorbidity Index as indicated by the presence of ICD-10 codes.^34^ We excluded enrollees with invalid state codes during 2019-2021 and dropped individuals residing in states that had adopted payment parity laws prior to 2020. We excluded enrollees with zero claims for all 12 months of a calendar year to make sure they are active users in the health plan.

##### **Supplemental Figure S2.2. State-level Telehealth Payment Parity Across States Pre and Post-Pandemic (by December, 2021)**

**
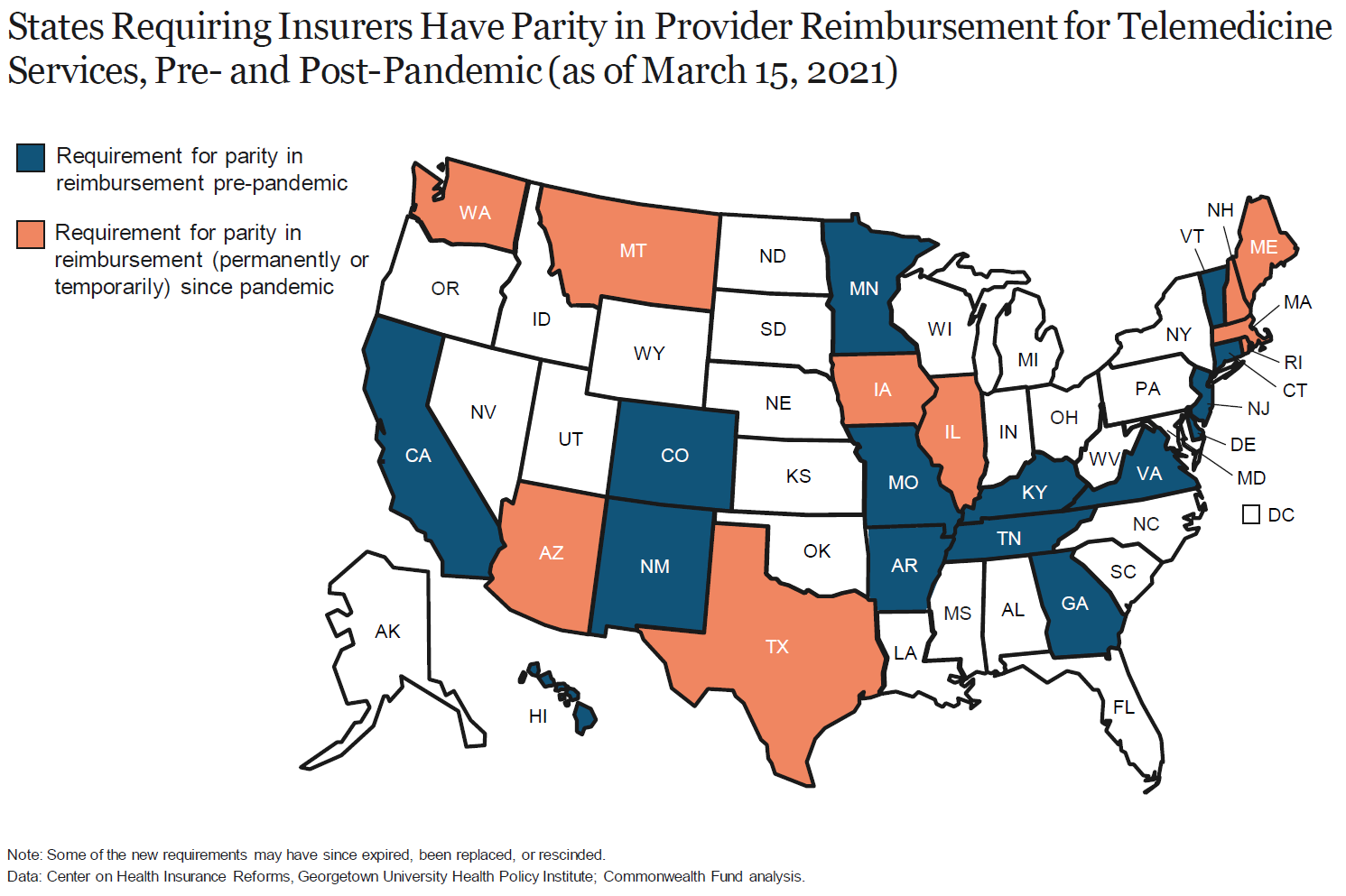
Source:** The original map is from the Center on Health Insurance Reforms, Georgetown University

Health Policy Institute, Commonwealth Fund Analysis.

Volk J, Palanker D, O’Brien M, L. Goe C. States’ Actions to Expand Telemedicine Access During COVID-19 and Future Policy Considerations. 2021. Accessed May 21, 2023. https://www.commonwealthfund.org/publications/issue-briefs/2021/jun/states-actions-expand-telemedicine-access-covid-19

##### **Supplemental Figure S2.3. States with Payment Parity by Year/Month**

| **Group** | **State** |  | **2019 Dec** | **2020 Mar/Apr** | **2021 Dec** |
| --- | --- | --- | --- | --- | --- |
| **Treated**  **(9 states with new parity after the onset of the pandemic)** | Arizona | AZ |  | ✓ | ✓ |
|  | Iowa | IA |  | ✓ | ✓ |
|  | Illinois | IL |  | ✓ | ✓ |
|  | Massachusetts | MA |  | ✓ | ✓ |
|  | Maine | ME |  | ✓ | ✓ |
|  | New Hampshire | NH |  | ✓ | ✓ |
|  | Rhode Island | RI |  | ✓ | ✓ |
|  | Washington | WA |  | ✓ | ✓ |
|  | Montana | MT |  | ✓ | ✓ |
| **Control**  **(25 states and DC, PR, VI without parity throughout the sample period)** | Alabama | AL |  |  |  |
|  | Alaska | AK |  |  |  |
|  | District of Columbia | DC |  |  |  |
|  | Florida | FL |  |  |  |
|  | Idaho | ID |  |  |  |
|  | Indiana | IN |  |  |  |
|  | Kansas | KS |  |  |  |
|  | Louisiana | LA |  |  |  |
|  | Maryland | MD |  |  |  |
|  | Michigan | MI |  |  |  |
|  | Mississippi | MS |  |  |  |
|  | Nebraska | NE |  |  |  |
|  | Nevada | NV |  |  |  |
|  | New York | NY |  |  |  |
|  | North Carolina | NC |  |  |  |
|  | North Dakota | ND |  |  |  |
|  | Ohio | OH |  |  |  |
|  | Oklahoma | OK |  |  |  |
|  | Oregon | OR |  |  |  |
|  | Pennsylvania | PA |  |  |  |
|  | Puerto Rico | PR |  |  |  |
|  | South Carolina | SC |  |  |  |
|  | South Dakota | SD |  |  |  |
|  | Utah | UT |  |  |  |
|  | Virgin Islands | VI |  |  |  |
|  | West Virginia | WV |  |  |  |
|  | Wisconsin | WI |  |  |  |
|  | Wyoming | WY |  |  |  |
| **Excluded**  **(16 states)** | Texas | TX |  | ✓ |  |
|  | 15 states (see notes) |  | ✓ | ✓ | ✓ |

**Note:** The treated group included individuals in the 9 states that moved to payment parity in response to the pandemic onset in March 2020 and continuously implemented the policy until the end of 2021. The control group comprised those individuals in 25 states, the District of Columbia (DC), Puerto Rico (PR), and Virgin Islands (VI) that had not implemented payment parity by 2021, either prior to or during the pandemic. In our main analysis, we excluded individuals in the 15 states (AR, CA, CO, CT, DE, GA, HI, KY, MN, MO, NJ, NM, TN, VA, VT) that exercised payment parity during pre and post the onset of the pandemic throughout the three-year period (the always group). We also excluded individuals in Texas, where payment parity was introduced during the pandemic but removed in December 2020.

##### **Supplemental Figure S2.4. The Number of Telehealth Visits across Service Types by Quarter, 2019-2021**

**
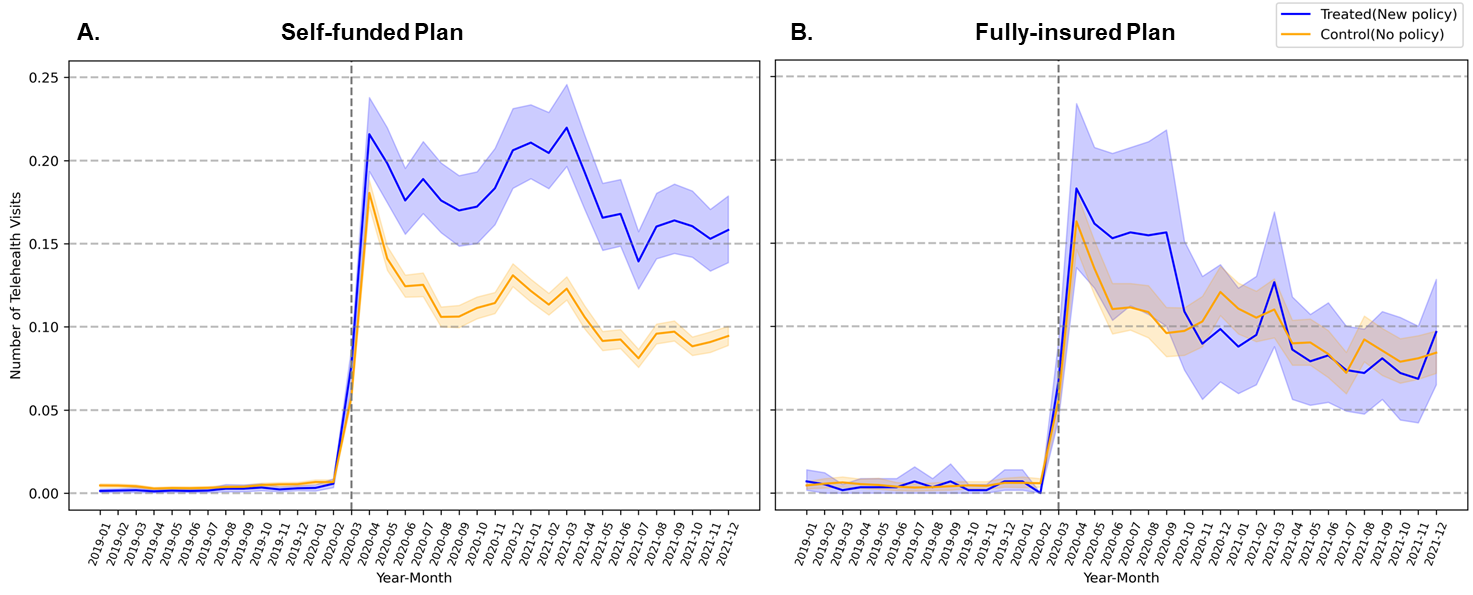
**

**Source:** Authors’ analysis of data from the Merative Commercial Claims and Encounters database, January 2019-December 2021.

**Notes:**

A. The analysis is based on the sample in self-funded plan with 890,640 person-month utilization observations.

B. The analysis is based on the sample in fully insured plan with 160,704 person-month utilization observations.

The blue line represents the number of telehealth visits by person by month in the treated group, while the orange line represents that in the control group.

##### **Supplemental Figure S2.5. Number of In-person Visits by Month, 2019-2021**

**
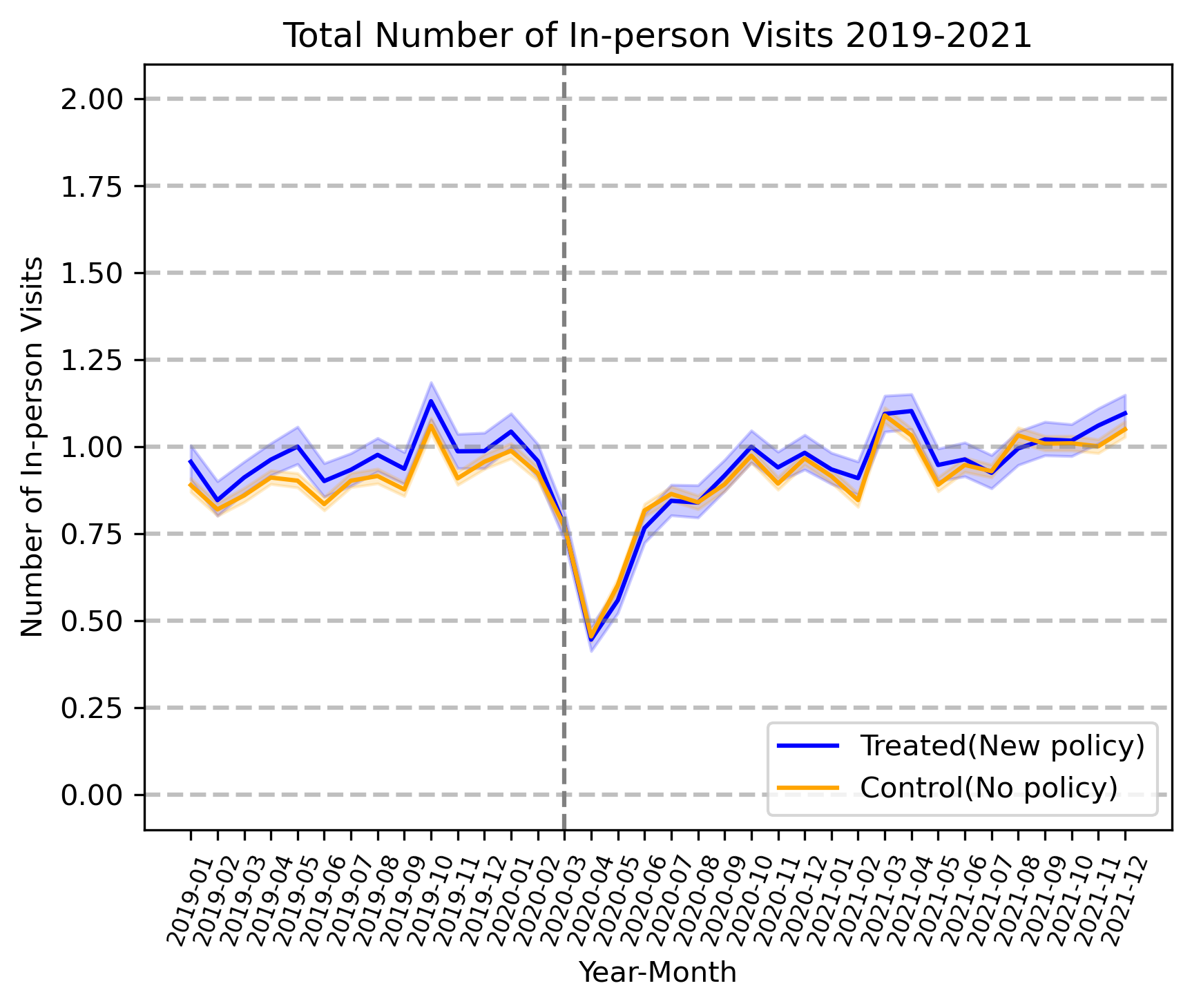
**

**Source:** Authors’ analysis of data from the Merative Commercial Claims and Encounters database, January 2019-December 2021.

**Notes:** The analysis was based on the full sample of 29,204 insured working-age adults with 1,051,344 person-month utilization observations. The blue line represents the number of in-person outpatient visits by person by month in the treated group, while the orange line represents that in the control group.

##### **Supplemental Figure S2.6. Total Number of Visits by Month, 2019-2021**


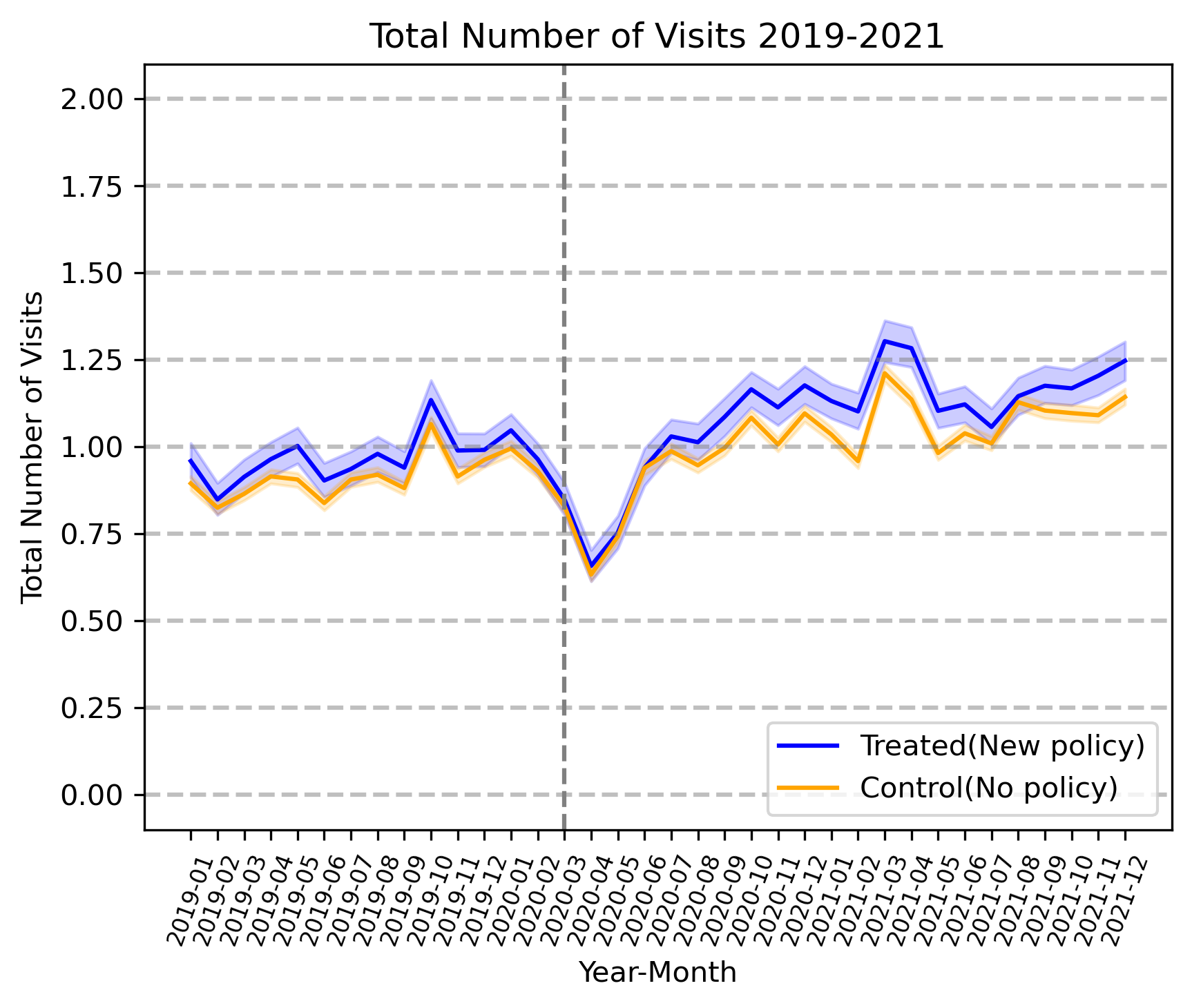


**Source:** Authors’ analysis of data from the Merative Commercial Claims and Encounters database, January 2019-December 2021.

**Notes:** The analysis was based on the full sample of 29,204 insured working-age adults with 1,051,344 person-month utilization observations. The blue line represents the total number of outpatient visits (in-person visits plus telehealth visits) by person by month in the treated group, while the orange line represents that in the control group.

##### **Supplemental Figure S2.7. Event Study: Number of Telehealth Visits by Month, 2019-2021**

**
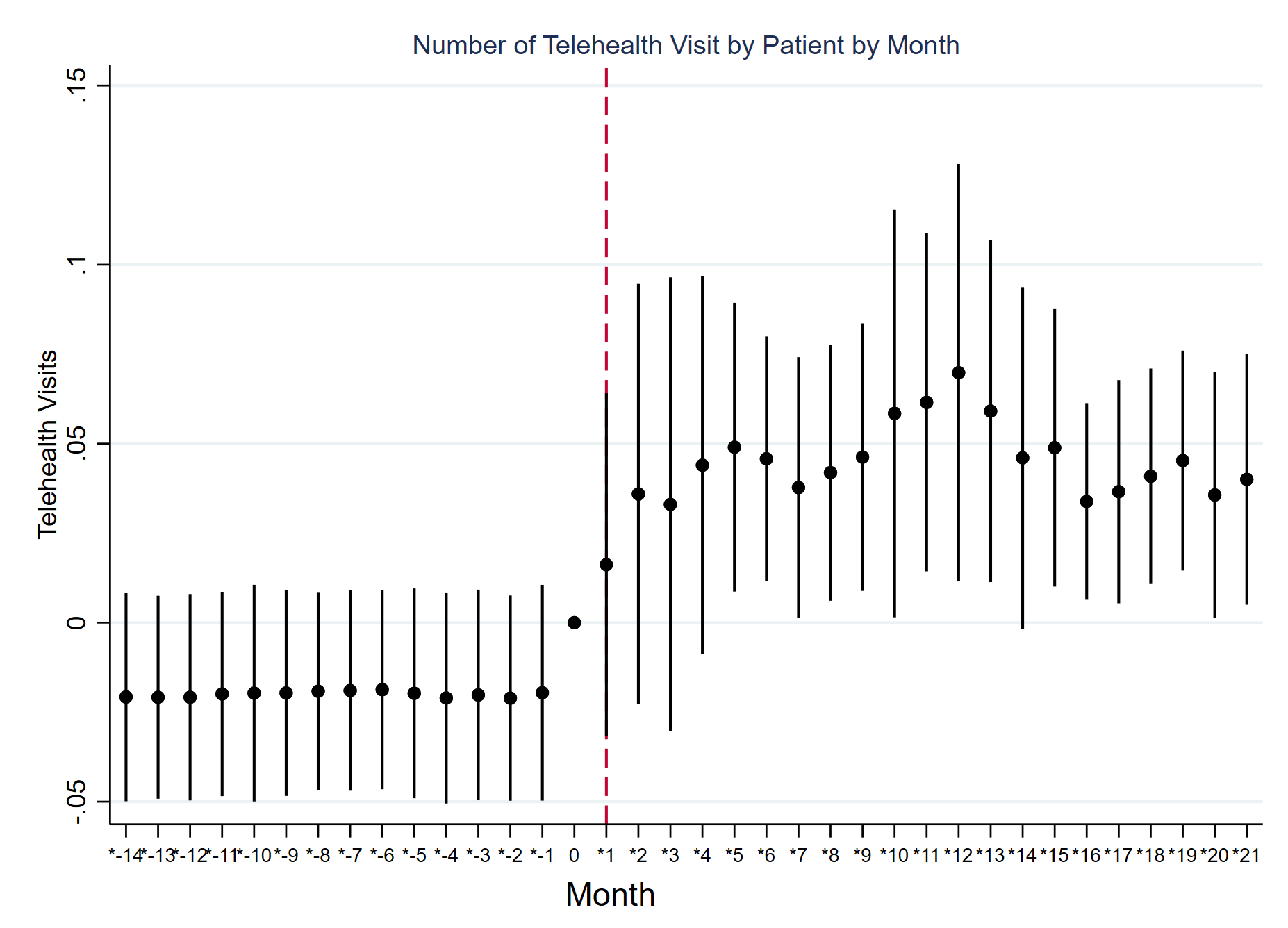
**

**Source:** Authors’ analysis of data from the Merative Commercial Claims and Encounters database, January 2019-December 2021.

###### **Supplemental Table S2.1. A List of Telehealth-Eligible Services and Codesa**

| **Type of Service** | **CPT and HCPCS Codes by Type of Service** |
| --- | --- |
| **Outpatient office** | 99201, 99202, 99203, 99204, 99205, 99211, 99212, 99213, 99214, 99215,  99354, 99355, 99421 c, 99422 c, 99423 c, 99441 c, 99442 c, 99443 c, G2012, G2061 c, G2062 c, G2063 c |
| **Behavioral health** | 0362T, 0373T, 90785, 90791, 90792, 90832, 90833, 90834, 90836, 90837, |
|  | 90838, 90839, 90840, 90845, 90846, 90847, 90853, 90875, 97151, 97152, |
|  | 97153, 97154, 97155, 97156, 97157, 97158, G0396, G0397, G0410, G0443,G2086, |
|  | G2087, G2088 |
| **Rehabilitation** | 97110, 97112, 97116, 97150, 97161, 97162, 97163, 97164, 97165, 97166,  97167, 97168, 97530, 97535, 97542, 97750, 97755, 97760, 97761, S9152 |
| **Other codes** | 77427, 90951, 90952, 90953, 90954, 90955 90956, 90957, 90958, 90959, |
|  | 90960, 90961, 90962, 90963, 90964, 90965, 90966, 90967, 90968, 90969, |
|  | 90970, 92002, 92004, 92012, 92014, 92507, 92508, 92521, 92522, 92523, |
|  | 92524, 92601, 92602, 92603, 92604, 94002, 94003, 94004, 94005, 94664, |
|  | 96110, 96112, 96113, 96116, 96121, 96127, 96130, 96131, 96132, 96133, |
|  | 96136, 96137, 96138, 96139, 96156, 96158, 96159, 96160, 96161, 96164, |
|  | 96165, 96167, 96168, 96170, 96171, 97802, 97803, 97804, 98966 c, 98967 c, |
|  | 98968 c, 98970 c, 98971 c, 98972 c, 99091 c, 99304, 99305, 99306, 99307, |
|  | 99308, 99309, 99310, 99315, 99316, 99324, 99325, 99326, 99327, 99328, |
|  | 99334, 99335, 99336, 99337, 99341, 99342, 99343, 99344, 99345, 99347, |
|  | 99348, 99349, 99350, 99406, 99407, 99446 c, 99447 c, 99448 c, 99449 c, |
|  | 99451 c, 99452 c, 99453 c, 99454 c, 99457 c, 99458 c, 99473, 99474 c, 99483, |
|  | 99495, 99496, 99497, 99498, G0108, G0109, G0270, G0296, G0406 c, G0407 c, |
|  | G0408 c, G0420, G0421, G0425 c, G0426 c, G0427 c,G0436, G0437,G0438, G0439, |
|  | G0442, G0444, G0445, G0446, G0447, G0459 c, G0506, |
|  | G0513, G0514, G2010 c, G9685, Q3014 c, T1014 c |
| a A list of CPT and HCPCS codes for telehealth-eligible services by type of service (source: CMS List of telehealth services. [https://www.cms.gov/Medicare/Medicare-General-Information/Telehealth/Telehealth-Codes)](https://www.cms.gov/Medicare/Medicare-General-Information/Telehealth/Telehealth-Codes)  c Code does not require a modifier or place of service code if provided via telehealth. | |

###### **Supplemental Table S2.2. Designating Telehealth Services and Type of Modality**

| **Code Type** | **Code** | **Definition** | **Modality Classification** |
| --- | --- | --- | --- |
| **Modifier** | GT | “Via interactive audio and video telecommunications systems.” | Video Supported telehealth |
|  | 95 | “Synchronous telemedicine service rendered via a real-time interactive audioand video telecommunications system.” | Video Supported- Telehealth |
| **Place of Service** | 02 | The location where health services and health-related services are provided or received, through a telecommunication system. | Telehealth - not specified |

**Assignment of Telehealth Eligible Service Codes to Specific Type of Virtual Encounter Modality**

| **Video supported telehealth** |
| --- |
| G0425, G0426, G0427, G0508, G0509 |
| **Telephone** |
| 98966, 98967, 98968, 99441, 99442, 99443 |

**Note:** Any telehealth encounter, based on having either a telehealth-specific CPT code or a telehealth modifier code, is referred to as a telehealth visit, henceforth.

###### **Supplemental Table S2.3. Summary Statistics: Characteristics of the Estimation Sample**

| **Characteristics** | **Full Sample**  **(person-months)** | **Pre-periods** | | **Post-periods** | |
| --- | --- | --- | --- | --- | --- |
|  | **All** | **Control** | **Treated** | **Control** | **Treated** |
|  | N=1,051,344 | N=364,395 | N=73,665 | N=510,153 | N=103,131 |
| **Dependent variables** |  |  |  |  |  |
| Probability of any telehealth visits | 0.049 (0.216) | 0.007 (0.083) | 0.006 (0.077) | 0.076 (0.265) | 0.098 (0.297) |
| Number of telehealth visits | 0.073 (0.405) | 0.008 (0.106) | 0.007 (0.113) | 0.110 (0.472) | 0.172 (0.696) |
| Number of in-person visits | 0.911 (1.634) | 0.902 (1.633) | 0.955 (1.727) | 0.908 (1.609) | 0.923 (1.691) |
| Number of total visits | 0.984 (1.716) | 0.910 (1.639) | 0.962 (1.734) | 1.018 (1.727) | 1.095 (1.890) |
| **Demographic characteristics** |  |  |  |  |  |
| Sex |  |  |  |  |  |
| Male | 456,048 (43.38%) | 157,770 (43.30%) | 32,250 (43.78%) | 220,878 (43.30%) | 45,150 (43.78%) |
| Female | 595,296 (56.62%) | 206,625 (56.70%) | 41,415 (56.22%) | 289,275 (56.70%) | 57,981 (56.22%) |
| Age (in years) | 45 (12) | 44 (12) | 44 (12) | 45 (12) | 45 (12) |
| Age group |  |  |  |  |  |
| 19-34 | 238,992 (22.73%) | 88,569 (24.31%) | 18,510 (25.13%) | 109,095 (21.38%) | 22,818 (22.13%) |
| 35-44 | 234,024 (22.26%) | 82,278 (22.58%) | 16,173 (21.95%) | 113,118 (22.17%) | 22,455 (21.77%) |
| 45-54 | 294,444 (28.01%) | 104,340 (28.63%) | 20,712 (28.12%) | 141,480 (27.73%) | 27,912 (27.06%) |
| 55-64 | 283,884 (27.00%) | 89,208 (24.48%) | 18,270 (24.80%) | 146,460 (28.71%) | 29,946 (29.04%) |
| **Socioeconomic characteristics** |  |  |  |  |  |
| Type of work |  |  |  |  |  |
| Intellectual | 372,276 (35.41%) | 128,025 (35.13%) | 27,090 (36.77%) | 179,235 (35.13%) | 37,926 (36.77%) |
| Manual | 404,748 (38.50%) | 136,200 (37.38%) | 32,445 (44.04%) | 190,680 (37.38%) | 45,423 (44.04%) |
| Other | 274,320 (26.09%) | 100,170 (27.49%) | 14,130 (19.18%) | 140,238 (27.49%) | 19,782 (19.18%) |
| Payroll type |  |  |  |  |  |
| Salary | 352,908 (33.57%) | 128,025 (35.13%) | 29,805 (40.46%) | 164,136 (32.17%) | 41,727 (40.46%) |
| Hourly | 285,732 (27.18%) | 136,200 (37.38%) | 21,900 (29.73%) | 136,017 (26.66%) | 30,660 (29.73%) |
| Unknown | 412,704 (39.25%) | 100,170 (27.49%) | 21,960 (29.81%) | 210,000 (41.16%) | 30,744 (29.81%) |
| Employer health plan indicator |  |  |  |  |  |
| Self-funded plans | 890,640 (84.71%) | 305,970 (83.97%) | 65,130 (88.41%) | 428,358 (83.97%) | 91,182 (88.41%) |
| Fully insured plans | 160,704 (15.29%) | 58,425 (16.03%) | 8,535 (11.59%) | 81,795 (16.03%) | 11,949 (11.59%) |
| Rurality |  |  |  |  |  |
| Rural | 202,536 (19.26%) | 75,690 (20.77%) | 8,700 (11.81%) | 105,966 (20.77%) | 12,180 (11.81%) |
| Urban | 848,808 (80.74%) | 288,705 (79.23%) | 64,965 (88.19%) | 404,187 (79.23%) | 90,951 (88.19%) |
| **Chronic health condition** |  |  |  |  |  |
| Comorbidity |  |  |  |  |  |
| Non-Chronic Condition | 622,080 (59.17%) | 212,835 (58.41%) | 64,911 (62.94%) | 297,969 (58.41%) | 64,911 (62.94%) |
| One Chronic Condition | 242,676 (23.08%) | 85,170 (23.37%) | 22,323 (21.65%) | 119,238 (23.37%) | 22,323 (21.65%) |
| Comorbidity | 186,588 (17.75%) | 66,390 (18.22%) | 15,897 (15.41%) | 92,946 (18.22%) | 15,897 (15.41%) |

###### Supplemental Table S2.4. DID Estimation: Health Service Utilization (Full Sample)

|  | **Probability of**  **Any telehealth visits** | **Number of**  **Telehealth visits** | **Number of**  **in-person visits** | **Number of**  **Total visits** |
| --- | --- | --- | --- | --- |
|  | b/se/ci95 | b/se/ci95 | b/se/ci95 | b/se/ci95 |
| **Treated** x **Post period** | 0.024^*^ | 0.064^**^ | -0.034 | 0.029^*^ |
|  | 0.013 | 0.030 | 0.025 | 0.015 |
|  | -0.001,0.049 | 0.004,0.124 | -0.085,0.016 | -0.002,0.060 |
| **Age** | -0.001^****^ | -0.002^****^ | 0.002^***^ | -0.001 |
|  | 0.000 | 0.000 | 0.000 | 0.001 |
|  | -0.001,-0.001 | -0.003,-0.002 | 0.001,0.003 | -0.002,0.001 |
| **Sex** |  |  |  |  |
| Male | Ref | Ref | Ref | Ref |
| Female | 0.013^****^ | 0.021^****^ | 0.184^****^ | 0.205^****^ |
|  | 0.001 | 0.003 | 0.010 | 0.012 |
|  | 0.010,0.016 | 0.016,0.027 | 0.163,0.204 | 0.181,0.230 |
| **Payroll type** |  |  |  |  |
| Salary | Ref | Ref | Ref | Ref |
| Hourly | -0.010^****^ | -0.021^****^ | -0.081^****^ | -0.102^****^ |
|  | 0.002 | 0.005 | 0.016 | 0.018 |
|  | -0.013,-0.007 | -0.032,-0.011 | -0.114,-0.048 | -0.138,-0.067 |
| Unknown | -0.002 | -0.013^*^ | 0.013 | 0.000 |
|  | 0.002 | 0.006 | 0.027 | 0.029 |
|  | -0.007,0.003 | -0.026,0.000 | -0.042,0.067 | -0.059,0.059 |
| **Type of work** |  |  |  |  |
| Intellectual work | Ref | Ref | Ref | Ref |
| Manual Work | -0.007^****^ | -0.016^****^ | 0.055^***^ | 0.038^**^ |
|  | 0.002 | 0.004 | 0.017 | 0.019 |
|  | -0.011,-0.004 | -0.024,-0.009 | 0.021,0.088 | 0.000,0.076 |
| Other | -0.004 | -0.008 | 0.101^**^ | 0.093^*^ |
|  | 0.004 | 0.006 | 0.048 | 0.050 |
|  | -0.012,0.003 | -0.019,0.004 | 0.005,0.197 | -0.008,0.195 |
| **Employer health plan indicator** |  |  |  |  |
| Self-funded plans | Ref | Ref | Ref | Ref |
| Fully insured plans | -0.009^*^ | -0.014^*^ | -0.154^***^ | -0.168^***^ |
|  | 0.005 | 0.008 | 0.053 | 0.057 |
|  | -0.019,0.001 | -0.030,0.002 | -0.260,-0.047 | -0.283,-0.053 |
| **Rurality** |  |  |  |  |
| Rural | Ref | Ref | Ref | Ref |
| Urban | 0.011^****^ | 0.019^***^ | 0.026^*^ | 0.046^**^ |
|  | 0.003 | 0.007 | 0.014 | 0.018 |
|  | 0.005,0.017 | 0.006,0.033 | -0.002,0.055 | 0.009,0.083 |
| **Comorbidity Index** | 0.022^****^ | 0.037^****^ | 0.353^****^ | 0.389^****^ |
|  | 0.001 | 0.004 | 0.014 | 0.016 |
|  | 0.019,0.024 | 0.030,0.044 | 0.324,0.381 | 0.357,0.422 |
| **Covid cases**  (per 1,000,000 population) | 0.022 | 0.022 | 0.073 | 0.094^*^ |
|  | 0.013 | 0.028 | 0.054 | 0.054 |
|  | -0.005,0.048 | -0.036,0.079 | -0.037,0.183 | -0.014,0.203 |
| Constant | 0.072^****^ | 0.135^****^ | 0.458^****^ | 0.593^****^ |
|  | 0.004 | 0.011 | 0.032 | 0.035 |
|  | 0.064,0.079 | 0.114,0.157 | 0.394,0.522 | 0.524,0.663 |
| Observations | 1051344 | 1051344 | 1051344 | 1051344 |

*p < 0.10 **p < 0.05 ***p < 0.01 ****p < 0.001

**Source:** Merative Commercial Claims and Encounters database 2019-2021

###### Supplemental Table S2.5. DID Estimation: Outpatient Service Utilization by Patient by Month (Self-funded health plans)

|  | **Probability of**  **Any telehealth visits** | **Number of**  **Telehealth visits** | **Number of**  **in-person visits** | **Number of**  **Total visits** |
| --- | --- | --- | --- | --- |
|  | b/se/ci95 | b/se/ci95 | b/se/ci95 | b/se/ci95 |
| **Treated** x **Post period** | 0.026^*^ | 0.070^**^ | -0.030 | 0.041^***^ |
|  | 0.013 | 0.032 | 0.029 | 0.015 |
|  | -0.001,0.052 | 0.006,0.135 | -0.088,0.029 | 0.010,0.071 |
| **Age** | -0.001^****^ | -0.002^****^ | 0.002^****^ | -0.000 |
|  | 0.000 | 0.000 | 0.000 | 0.001 |
|  | -0.001,-0.001 | -0.003,-0.002 | 0.001,0.003 | -0.002,0.001 |
| **Sex** |  |  |  |  |
| Male | Ref | Ref | Ref | Ref |
| Female | 0.013^****^ | 0.022^****^ | 0.187^****^ | 0.209^****^ |
|  | 0.002 | 0.003 | 0.009 | 0.011 |
|  | 0.010,0.016 | 0.016,0.029 | 0.168,0.206 | 0.186,0.232 |
| **Payroll type** |  |  |  |  |
| Salary | Ref | Ref | Ref | Ref |
| Hourly | -0.010^****^ | -0.022^****^ | -0.078^****^ | -0.100^****^ |
|  | 0.002 | 0.005 | 0.017 | 0.018 |
|  | -0.013,-0.007 | -0.032,-0.011 | -0.112,-0.045 | -0.136,-0.064 |
| Unknown | -0.002 | -0.013^*^ | 0.015 | 0.002 |
|  | 0.002 | 0.006 | 0.027 | 0.029 |
|  | -0.007,0.003 | -0.025,0.000 | -0.040,0.069 | -0.057,0.061 |
| **Type of work** |  |  |  |  |
| Intellectual work | Ref | Ref | Ref | Ref |
| Manual Work | -0.008^****^ | -0.017^****^ | 0.056^***^ | 0.039^**^ |
|  | 0.002 | 0.004 | 0.017 | 0.019 |
|  | -0.011,-0.004 | -0.024,-0.009 | 0.022,0.090 | 0.001,0.077 |
| Other | -0.005 | -0.008 | 0.102^**^ | 0.093^*^ |
|  | 0.004 | 0.005 | 0.049 | 0.051 |
|  | -0.012,0.002 | -0.019,0.003 | 0.004,0.199 | -0.009,0.195 |
| **Rurality** |  |  |  |  |
| Rural | Ref | Ref | Ref | Ref |
| Urban |  |  |  |  |
|  | 0.010^***^ | 0.018^**^ | 0.023 | 0.041^*^ |
|  | 0.003 | 0.007 | 0.017 | 0.020 |
|  | 0.004,0.016 | 0.003,0.032 | -0.011,0.056 | -0.000,0.082 |
| **Comorbidity Index** |  |  |  |  |
|  | 0.023^****^ | 0.038^****^ | 0.342^****^ | 0.380^****^ |
|  | 0.001 | 0.004 | 0.010 | 0.011 |
|  | 0.020,0.025 | 0.031,0.046 | 0.322,0.361 | 0.357,0.403 |
| **Covid cases**  (per 1,000,000 population) | 0.021 | 0.017 | 0.063 | 0.080 |
|  | 0.013 | 0.028 | 0.058 | 0.056 |
|  | -0.005,0.046 | -0.040,0.074 | -0.054,0.180 | -0.033,0.193 |
| Constant | 0.074^****^ | 0.141^****^ | 0.440^****^ | 0.581^****^ |
|  | 0.004 | 0.011 | 0.035 | 0.039 |
|  | 0.066,0.082 | 0.118,0.164 | 0.369,0.511 | 0.502,0.660 |
| Observations | 890640 | 890640 | 890640 | 890640 |

*p < 0.10 **p < 0.05 ***p < 0.01 ****p < 0.001

**Source:** Merative Commercial Claims and Encounters database 2019-2021

###### Supplemental Table S2.6. DID Estimation: Outpatient Service Utilization (Fully insured health plans)

|  | **Probability of**  **Any telehealth visits** | **Number of**  **Telehealth visits** | **Number of**  **in-person visits** | **Number of**  **Total visits** |
| --- | --- | --- | --- | --- |
|  | b/se/ci95 | b/se/ci95 | b/se/ci95 | b/se/ci95 |
| **Treated** x **Post period** | 0.008 | 0.009 | -0.054 | -0.045 |
|  | 0.010 | 0.019 | 0.035 | 0.040 |
|  | -0.012,0.027 | -0.029,0.047 | -0.124,0.015 | -0.125,0.035 |
| **Age** | -0.001^****^ | -0.001^***^ | -0.001 | -0.002 |
|  | 0.000 | 0.000 | 0.002 | 0.002 |
|  | -0.001,-0.000 | -0.002,-0.001 | -0.004,0.003 | -0.006,0.002 |
| **Sex** |  |  |  |  |
| male | Ref | Ref | Ref | Ref |
| female | 0.011^****^ | 0.017^****^ | 0.160^****^ | 0.176^****^ |
|  | 0.002 | 0.005 | 0.031 | 0.033 |
|  | 0.006,0.016 | 0.007,0.026 | 0.097,0.222 | 0.110,0.242 |
| **Rurality** |  |  |  |  |
| Rural | Ref | Ref | Ref | Ref |
| Urban | 0.015^****^ | 0.027^***^ | 0.059 | 0.086^*^ |
|  | 0.004 | 0.008 | 0.040 | 0.044 |
|  | 0.007,0.022 | 0.010,0.044 | -0.021,0.139 | -0.003,0.175 |
| **Comorbidity Index** | Ref | Ref | Ref | Ref |
|  | 0.016^****^ | 0.029^****^ | 0.411^****^ | 0.440^****^ |
|  | 0.001 | 0.004 | 0.045 | 0.047 |
|  | 0.013,0.019 | 0.020,0.038 | 0.320,0.502 | 0.345,0.536 |
| **Covid cases**  (per 1,000,000 population) | 0.028 | 0.045 | 0.118 | 0.163 |
|  | 0.025 | 0.046 | 0.087 | 0.103 |
|  | -0.023,0.078 | -0.049,0.139 | -0.057,0.293 | -0.045,0.371 |
| Constant | 0.041^****^ | 0.070^****^ | 0.512^****^ | 0.582^****^ |
|  | 0.005 | 0.012 | 0.075 | 0.079 |
|  | 0.031,0.051 | 0.047,0.093 | 0.361,0.663 | 0.423,0.742 |
| Observations | 160704 | 160704 | 160704 | 160704 |

*p < 0.10 **p < 0.05 ***p < 0.01 ****p < 0.001

**Source:** Merative Commercial Claims and Encounters database 2019-2021

###### Supplemental Table S2.7. DID Estimation: Probability of Any Telehealth Visit

|  | **Model1** | **Model2** | **Model3** | **Model4** | **Model5** | **Model6** |
| --- | --- | --- | --- | --- | --- | --- |
|  | b/se/ci95 | b/se/ci95 | b/se/ci95 | b/se/ci95 | b/se/ci95 | b/se/ci95 |
| **Treated** x **Post period** | 0.023^*^ | 0.023^*^ | 0.023^*^ | 0.023^*^ | 0.024^*^ | 0.024^*^ |
|  | 0.012 | 0.012 | 0.012 | 0.012 | 0.013 | 0.013 |
|  | -0.002,0.048 | -0.002,0.048 | -0.002,0.048 | -0.002,0.048 | -0.001,0.049 | -0.001,0.049 |
| **Age** |  | -0.001^****^ | -0.001^****^ | -0.001^****^ | -0.001^****^ | -0.001^****^ |
|  |  | 0.000 | 0.000 | 0.000 | 0.000 | 0.000 |
|  |  | -0.002,-0.001 | -0.002,-0.001 | -0.001,-0.001 | -0.001,-0.001 | -0.001,-0.001 |
| **Sex** |  |  |  |  |  |  |
| Male |  | Ref | Ref | Ref | Ref | Ref |
| Female |  | 0.014^****^ | 0.014^****^ | 0.013^****^ | 0.013^****^ | 0.013^****^ |
|  |  | 0.001 | 0.002 | 0.001 | 0.001 | 0.001 |
|  |  | 0.011,0.017 | 0.011,0.017 | 0.010,0.016 | 0.010,0.016 | 0.010,0.016 |
| **Payroll type** |  |  |  |  |  |  |
| Salary |  |  | Ref | Ref | Ref | Ref |
| Hourly |  |  | -0.006^****^ | -0.010^****^ | -0.010^****^ | -0.010^****^ |
|  |  |  | 0.002 | 0.002 | 0.002 | 0.002 |
|  |  |  | -0.010,-0.003 | -0.013,-0.007 | -0.013,-0.007 | -0.013,-0.007 |
| Unknown |  |  | -0.000 | -0.002 | -0.002 | -0.002 |
|  |  |  | 0.003 | 0.002 | 0.002 | 0.002 |
|  |  |  | -0.005,0.005 | -0.007,0.003 | -0.007,0.003 | -0.007,0.003 |
| **Type of work** |  |  |  |  |  |  |
| Intellectual work |  |  | Ref | Ref | Ref | Ref |
| Manual Work |  |  | -0.007^****^ | -0.007^****^ | -0.007^****^ | -0.007^****^ |
|  |  |  | 0.002 | 0.002 | 0.002 | 0.002 |
|  |  |  | -0.010,-0.004 | -0.011,-0.004 | -0.011,-0.004 | -0.011,-0.004 |
| Other |  |  | -0.004 | -0.004 | -0.004 | -0.004 |
|  |  |  | 0.004 | 0.004 | 0.004 | 0.004 |
|  |  |  | -0.012,0.005 | -0.012,0.003 | -0.012,0.003 | -0.012,0.003 |
| **Employer health plan indicator** |  |  |  |  |  |  |
| Self-funded plans |  |  | Ref | Ref | Ref | Ref |
| Fully insured plans |  |  | -0.008 | -0.009^*^ | -0.009^*^ | -0.009^*^ |
|  |  |  | 0.005 | 0.005 | 0.005 | 0.005 |
|  |  |  | -0.019,0.002 | -0.019,0.001 | -0.019,0.001 | -0.019,0.001 |
| **Rurality** |  |  |  |  |  |  |
| Rural |  |  |  |  |  |  |
| Urban |  |  | 0.010^***^ | 0.011^****^ | 0.011^****^ | 0.011^****^ |
|  |  |  | 0.003 | 0.003 | 0.003 | 0.003 |
|  |  |  | 0.004,0.016 | 0.005,0.017 | 0.005,0.017 | 0.005,0.016 |
| **Comorbidity Index** |  |  |  |  |  |  |
|  |  |  |  | 0.022^****^ | 0.022^****^ | 0.022^****^ |
|  |  |  |  | 0.001 | 0.001 | 0.001 |
|  |  |  |  | 0.019,0.024 | 0.019,0.024 | 0.019,0.024 |
| **Covid cases**  (per 1,000,000 population) |  |  |  |  | 0.022^****^ | 0.022^****^ |
|  |  |  |  |  | 0.013 | 0.001 |
|  |  |  |  |  | -0.005,0.048 | 0.019,0.024 |
| **Region** |  |  |  |  |  |  |
| Northeast |  |  |  |  |  | Ref |
| North Central |  |  |  |  |  | -0.036^***^ |
|  |  |  |  |  |  | 0.012 |
|  |  |  |  |  |  | -0.059,-0.012 |
| South |  |  |  |  |  | -0.030^***^ |
|  |  |  |  |  |  | 0.009 |
|  |  |  |  |  |  | -0.048,-0.012 |
| West |  |  |  |  |  | -0.025^**^ |
|  |  |  |  |  |  | 0.011 |
|  |  |  |  |  |  | -0.046,-0.003 |
| Constant | 0.049^****^ | 0.061^****^ | 0.060^****^ | 0.073^****^ | 0.072^****^ | 0.097^****^ |
|  | 0.001 | 0.004 | 0.004 | 0.004 | 0.004 | 0.009 |
|  | 0.047,0.052 | 0.053,0.070 | 0.051,0.068 | 0.065,0.081 | 0.064,0.079 | 0.079,0.115 |
| Observations | 1051344 | 1051344 | 1051344 | 1051344 | 1051344 | 1051344 |

*p < 0.10 **p < 0.05 ***p < 0.01 ****p < 0.001 **Source:** Merative Commercial Claims and Encounters database 2019-2021

###### Supplemental Table S2.8. DID Estimation: Number of Telehealth Visits

|  | **Model1** | **Model2** | **Model3** | **Model4** | **Model5** | **Model6** |
| --- | --- | --- | --- | --- | --- | --- |
|  | b/se/ci95 | b/se/ci95 | b/se/ci95 | b/se/ci95 | b/se/ci95 | b/se/ci95 |
| **Treated** x **Post period** | 0.063^**^ | 0.063^**^ | 0.063^**^ | 0.063^**^ | 0.064^**^ | 0.064^**^ |
|  | 0.030 | 0.030 | 0.030 | 0.030 | 0.030 | 0.030 |
|  | 0.003,0.123 | 0.003,0.123 | 0.003,0.123 | 0.003,0.123 | 0.004,0.124 | 0.004,0.124 |
| **Age** |  | -0.001^****^ | -0.001^****^ | -0.002^****^ | -0.002^****^ | -0.002^****^ |
|  |  | 0.000 | 0.000 | 0.000 | 0.000 | 0.000 |
|  |  | -0.002,-0.001 | -0.002,-0.001 | -0.003,-0.002 | -0.003,-0.002 | -0.003,-0.002 |
| **Sex** |  |  |  |  |  |  |
| Male |  | Ref | Ref | Ref | Ref | Ref |
| Female |  | 0.024^****^ | 0.023^****^ | 0.021^****^ | 0.021^****^ | 0.021^****^ |
|  |  | 0.003 | 0.003 | 0.003 | 0.003 | 0.003 |
|  |  | 0.017,0.030 | 0.016,0.029 | 0.016,0.027 | 0.016,0.027 | 0.016,0.027 |
| **Payroll type** |  |  |  |  |  |  |
| Salary |  |  | Ref | Ref | Ref | Ref |
| Hourly |  |  | -0.015^***^ | -0.021^****^ | -0.021^****^ | -0.021^****^ |
|  |  |  | 0.005 | 0.005 | 0.005 | 0.005 |
|  |  |  | -0.025,-0.005 | -0.032,-0.011 | -0.032,-0.011 | -0.032,-0.011 |
| Unknown |  |  | -0.009 | -0.013^*^ | -0.013^*^ | -0.013^*^ |
|  |  |  | 0.006 | 0.006 | 0.006 | 0.006 |
|  |  |  | -0.022,0.003 | -0.026,0.000 | -0.026,0.000 | -0.026,0.000 |
| **Type of work** |  |  |  |  |  |  |
| Intellectual work |  |  | Ref | Ref | Ref | Ref |
| Manual Work |  |  | -0.016^****^ | -0.016^****^ | -0.016^****^ | -0.016^****^ |
|  |  |  | 0.004 | 0.004 | 0.004 | 0.004 |
|  |  |  | -0.023,-0.008 | -0.024,-0.009 | -0.024,-0.009 | -0.024,-0.009 |
| Other |  |  | -0.006 | -0.008 | -0.008 | -0.007 |
|  |  |  | 0.006 | 0.006 | 0.006 | 0.006 |
|  |  |  | -0.019,0.006 | -0.019,0.004 | -0.019,0.004 | -0.019,0.004 |
| **Employer health plan indicator** |  |  |  |  |  |  |
| Self-funded plans |  |  | Ref | Ref | Ref | Ref |
| Fully insured plans |  |  | -0.013 | -0.014^*^ | -0.014^*^ | -0.014^*^ |
|  |  |  | 0.008 | 0.008 | 0.008 | 0.008 |
|  |  |  | -0.029,0.003 | -0.030,0.002 | -0.030,0.002 | -0.030,0.002 |
| **Rurality** |  |  |  |  |  |  |
| Rural |  |  | Ref | Ref | Ref | Ref |
| Urban |  |  | 0.018^**^ | 0.019^***^ | 0.019^***^ | 0.019^***^ |
|  |  |  | 0.007 | 0.007 | 0.007 | 0.007 |
|  |  |  | 0.004,0.032 | 0.006,0.033 | 0.006,0.033 | 0.006,0.033 |
| **Comorbidity Index** |  |  |  |  |  |  |
|  |  |  |  | 0.037^****^ | 0.037^****^ | 0.037^****^ |
|  |  |  |  | 0.004 | 0.004 | 0.004 |
|  |  |  |  | 0.030,0.044 | 0.030,0.044 | 0.030,0.044 |
| **Covid cases**  (per 1,000,000 population) |  |  |  |  | 0.022 | 0.021 |
|  |  |  |  |  | 0.028 | 0.028 |
|  |  |  |  |  | -0.036,0.079 | -0.036,0.079 |
| **Region** |  |  |  |  |  |  |
| Northeast |  |  |  |  |  | Ref |
| North Central |  |  |  |  |  | -0.046^**^ |
|  |  |  |  |  |  | 0.019 |
|  |  |  |  |  |  | -0.085,-0.007 |
| South |  |  |  |  |  | -0.043^***^ |
|  |  |  |  |  |  | 0.015 |
|  |  |  |  |  |  | -0.073,-0.014 |
| West |  |  |  |  |  | -0.014 |
|  |  |  |  |  |  | 0.020 |
|  |  |  |  |  |  | -0.054,0.025 |
| Constant | 0.074^****^ | 0.113^****^ | 0.114^****^ | 0.137^****^ | 0.135^****^ | 0.169^****^ |
|  | 0.004 | 0.011 | 0.011 | 0.011 | 0.011 | 0.017 |
|  | 0.066,0.082 | 0.090,0.135 | 0.092,0.136 | 0.114,0.159 | 0.114,0.157 | 0.134,0.204 |
| Observations | 1051344 | 1051344 | 1051344 | 1051344 | 1051344 | 1051344 |

*p < 0.10 **p < 0.05 ***p < 0.01 ****p < 0.001 **Source:** Merative Commercial Claims and Encounters database 2019-2021

| **Supplemental Table S2.9. DID Estimation: Number of In-person Visits** | | | | | | |
| --- | --- | --- | --- | --- | --- | --- |
|  | **Model1** | **Model2** | **Model3** | **Model4** | **Model5** | **Model6** |
|  | b/se/ci95 | b/se/ci95 | b/se/ci95 | b/se/ci95 | b/se/ci95 | b/se/ci95 |
| **Treated** x **Post period** | -0.038 | -0.038 | -0.038 | -0.038 | -0.034 | -0.034 |
|  | 0.025 | 0.025 | 0.025 | 0.025 | 0.025 | 0.025 |
|  | -0.088,0.012 | -0.088,0.012 | -0.088,0.012 | -0.088,0.012 | -0.085,0.016 | -0.085,0.016 |
| **Age** |  | 0.011^****^ | 0.011^****^ | 0.002^***^ | 0.002^***^ | 0.002^***^ |
|  |  | 0.000 | 0.000 | 0.000 | 0.000 | 0.000 |
|  |  | 0.011,0.012 | 0.010,0.012 | 0.001,0.003 | 0.001,0.003 | 0.001,0.003 |
| **Sex** |  |  |  |  |  |  |
| Male |  | Ref | Ref | Ref | Ref | Ref |
| Female |  | 0.190^****^ | 0.194^****^ | 0.184^****^ | 0.184^****^ | 0.184^****^ |
|  |  | 0.012 | 0.012 | 0.010 | 0.010 | 0.010 |
|  |  | 0.166,0.214 | 0.169,0.219 | 0.163,0.204 | 0.163,0.204 | 0.163,0.204 |
| **Payroll type** |  |  |  |  |  |  |
| Salary |  |  | Ref | Ref | Ref | Ref |
| Hourly |  |  | -0.024 | -0.081^****^ | -0.081^****^ | -0.081^****^ |
|  |  |  | 0.016 | 0.016 | 0.016 | 0.016 |
|  |  |  | -0.055,0.008 | -0.114,-0.048 | -0.114,-0.048 | -0.113,-0.048 |
| Unknown |  |  | 0.042 | 0.013 | 0.013 | 0.013 |
|  |  |  | 0.031 | 0.027 | 0.027 | 0.027 |
|  |  |  | -0.020,0.104 | -0.042,0.067 | -0.042,0.067 | -0.042,0.067 |
| **Type of work** |  |  |  |  |  |  |
| Intellectual work |  |  | Ref | Ref | Ref | Ref |
| Manual Work |  |  | 0.062^****^ | 0.055^***^ | 0.055^***^ | 0.055^***^ |
|  |  |  | 0.017 | 0.017 | 0.017 | 0.017 |
|  |  |  | 0.028,0.096 | 0.021,0.088 | 0.021,0.088 | 0.021,0.088 |
| Other |  |  | 0.113^**^ | 0.101^**^ | 0.101^**^ | 0.101^**^ |
|  |  |  | 0.054 | 0.048 | 0.048 | 0.048 |
|  |  |  | 0.005,0.221 | 0.005,0.197 | 0.005,0.197 | 0.005,0.197 |
| **Employer health plan indicator** |  |  |  |  |  |  |
| Self-funded plans |  |  | Ref | Ref | Ref | Ref |
| Fully insured plans |  |  | -0.144^**^ | -0.153^***^ | -0.154^***^ | -0.153^***^ |
|  |  |  | 0.059 | 0.053 | 0.053 | 0.053 |
|  |  |  | -0.262,-0.026 | -0.260,-0.047 | -0.260,-0.047 | -0.260,-0.047 |
| **Rurality** |  |  |  |  |  |  |
| Rural |  |  | Ref | Ref | Ref | Ref |
| Urban |  |  | 0.015 | 0.026^*^ | 0.026^*^ | 0.026^*^ |
|  |  |  | 0.016 | 0.014 | 0.014 | 0.014 |
|  |  |  | -0.017,0.047 | -0.002,0.055 | -0.002,0.055 | -0.002,0.055 |
| **Comorbidity Index** |  |  |  |  |  |  |
|  |  |  |  | 0.353^****^ | 0.353^****^ | 0.353^****^ |
|  |  |  |  | 0.014 | 0.014 | 0.014 |
|  |  |  |  | 0.324,0.381 | 0.324,0.381 | 0.324,0.382 |
| **Covid cases**  (per 1,000,000 population) |  |  |  |  | 0.073 | 0.073 |
|  |  |  |  |  | 0.054 | 0.054 |
|  |  |  |  |  | -0.037,0.183 | -0.037,0.182 |
| **Region** |  |  |  |  |  |  |
| Northeast |  |  |  |  |  | Ref |
| North Central |  |  |  |  |  | 0.012 |
|  |  |  |  |  |  | 0.109 |
|  |  |  |  |  |  | -0.206,0.231 |
| South |  |  |  |  |  | -0.047 |
|  |  |  |  |  |  | 0.095 |
|  |  |  |  |  |  | -0.239,0.145 |
| West |  |  |  |  |  | 0.018 |
|  |  |  |  |  |  | 0.150 |
|  |  |  |  |  |  | -0.284,0.321 |
| Constant | 0.912^****^ | 0.293^****^ | 0.248^****^ | 0.463^****^ | 0.458^****^ | 0.477^****^ |
|  | 0.016 | 0.022 | 0.033 | 0.032 | 0.032 | 0.088 |
|  | 0.881,0.944 | 0.248,0.339 | 0.182,0.314 | 0.398,0.528 | 0.394,0.522 | 0.301,0.653 |
| Observations | 1051344 | 1051344 | 1051344 | 1051344 | 1051344 | 1051344 |

*p < 0.10 **p < 0.05 ***p < 0.01 ****p < 0.001

**Source:** Merative Commercial Claims and Encounters databaseData 2019-2021

###### Supplemental Table S2.10. DID Estimation: Total Number of Visits

|  | **Model1** | **Model2** | **Model3** | **Model4** | **Model5** | **Model6** |
| --- | --- | --- | --- | --- | --- | --- |
|  | b/se/ci95 | b/se/ci95 | b/se/ci95 | b/se/ci95 | b/se/ci95 | b/se/ci95 |
| **Treated** x **Post period** | 0.025 | 0.025 | 0.025 | 0.025 | 0.029^*^ | 0.029^*^ |
|  | 0.015 | 0.015 | 0.015 | 0.015 | 0.015 | 0.015 |
|  | -0.005,0.055 | -0.005,0.055 | -0.005,0.055 | -0.005,0.055 | -0.002,0.060 | -0.002,0.060 |
| **Age** |  | 0.010^****^ | 0.010^****^ | -0.001 | -0.001 | -0.001 |
|  |  | 0.001 | 0.001 | 0.001 | 0.001 | 0.001 |
|  |  | 0.009,0.012 | 0.009,0.011 | -0.002,0.001 | -0.002,0.001 | -0.002,0.001 |
| **Sex** |  |  |  |  |  |  |
| Male |  | Ref | Ref | Ref | Ref | Ref |
| Female |  | 0.214^****^ | 0.216^****^ | 0.205^****^ | 0.205^****^ | 0.205^****^ |
|  |  | 0.014 | 0.015 | 0.012 | 0.012 | 0.012 |
|  |  | 0.186,0.242 | 0.187,0.246 | 0.181,0.230 | 0.181,0.230 | 0.181,0.229 |
| **Payroll type** |  |  |  |  |  |  |
| Salary |  |  | Ref | Ref | Ref | Ref |
| Hourly |  |  | -0.039^**^ | -0.102^****^ | -0.102^****^ | -0.102^****^ |
|  |  |  | 0.017 | 0.018 | 0.018 | 0.017 |
|  |  |  | -0.073,-0.005 | -0.138,-0.067 | -0.138,-0.067 | -0.137,-0.067 |
| Unknown |  |  | 0.033 | -0.000 | 0.000 | -0.000 |
|  |  |  | 0.034 | 0.029 | 0.029 | 0.029 |
|  |  |  | -0.035,0.100 | -0.059,0.059 | -0.059,0.059 | -0.059,0.059 |
| **Type of work** |  |  |  |  |  |  |
| Intellectual work |  |  | Ref | Ref | Ref | Ref |
| Manual Work |  |  | 0.047^**^ | 0.038^**^ | 0.038^**^ | 0.038^**^ |
|  |  |  | 0.019 | 0.019 | 0.019 | 0.019 |
|  |  |  | 0.008,0.085 | 0.000,0.076 | 0.000,0.076 | 0.000,0.076 |
| Other |  |  | 0.107^*^ | 0.093^*^ | 0.093^*^ | 0.094^*^ |
|  |  |  | 0.057 | 0.050 | 0.050 | 0.050 |
|  |  |  | -0.008,0.221 | -0.008,0.195 | -0.008,0.195 | -0.008,0.195 |
| **Employer health plan indicator** |  |  |  |  |  |  |
| Self-funded plans |  |  | Ref | Ref | Ref | Ref |
| Fully insured plans |  |  | -0.157^**^ | -0.168^***^ | -0.168^***^ | -0.167^***^ |
|  |  |  | 0.063 | 0.057 | 0.057 | 0.057 |
|  |  |  | -0.284,-0.030 | -0.282,-0.053 | -0.283,-0.053 | -0.282,-0.053 |
| **Rurality** |  |  |  |  |  |  |
| Rural |  |  | Ref | Ref | Ref | Ref |
| Urban |  |  | 0.033 | 0.046^**^ | 0.046^**^ | 0.046^**^ |
|  |  |  | 0.021 | 0.018 | 0.018 | 0.018 |
|  |  |  | -0.009,0.074 | 0.009,0.083 | 0.009,0.083 | 0.009,0.083 |
| **Comorbidity Index** |  |  |  | 0.389^****^ | 0.389^****^ | 0.390^****^ |
|  |  |  |  | 0.016 | 0.016 | 0.016 |
|  |  |  |  | 0.357,0.422 | 0.357,0.422 | 0.357,0.422 |
| **Covid cases**  (per 1,000,000 population) |  |  |  |  | 0.094^*^ | 0.094^*^ |
|  |  |  |  |  | 0.054 | 0.054 |
|  |  |  |  |  | -0.014,0.203 | -0.014,0.203 |
| **Region** |  |  |  |  |  |  |
| Northeast |  |  |  |  |  | Ref |
| North Central |  |  |  |  |  | -0.034 |
|  |  |  |  |  |  | 0.118 |
|  |  |  |  |  |  | -0.272,0.205 |
| South |  |  |  |  |  | -0.090 |
|  |  |  |  |  |  | 0.095 |
|  |  |  |  |  |  | -0.281,0.101 |
| West |  |  |  |  |  | 0.004 |
|  |  |  |  |  |  | 0.158 |
|  |  |  |  |  |  | -0.314,0.322 |
| Constant | 0.986^****^ | 0.406^****^ | 0.363^****^ | 0.600^****^ | 0.593^****^ | 0.646^****^ |
|  | 0.018 | 0.029 | 0.038 | 0.036 | 0.035 | 0.088 |
|  | 0.951,1.022 | 0.347,0.465 | 0.286,0.439 | 0.528,0.671 | 0.524,0.663 | 0.468,0.823 |
| Observations | 1051344 | 1051344 | 1051344 | 1051344 | 1051344 | 1051344 |

*p < 0.10 **p < 0.05 ***p < 0.01 ****p < 0.001 **Source:** Merative Commercial Claims and Encounters database 2019-2021

###### Supplemental Table S2.11. DID Estimation: Outpatient Service Utilization among Individuals in Treated and New Control Group (Control plus Always Group)

|  | **Probability of**  **Any telehealth visits** | **Number of**  **Telehealth visits** | **Number of**  **in-person visits** | **Number of**  **Total visits** |
| --- | --- | --- | --- | --- |
|  | b/se/ci95 | b/se/ci95 | b/se/ci95 | b/se/ci95 |
| **Treated** x **Post period** | 0.021 | 0.058^*^ | -0.032 | 0.026^*^ |
|  | 0.013 | 0.030 | 0.024 | 0.014 |
|  | -0.005,0.046 | -0.002,0.117 | -0.081,0.016 | -0.003,0.054 |
| **Age** | -0.001^****^ | -0.002^****^ | 0.001^****^ | -0.001 |
|  | 0.000 | 0.000 | 0.000 | 0.000 |
|  | -0.001,-0.001 | -0.003,-0.002 | 0.001,0.002 | -0.002,0.000 |
| **Sex** |  |  |  |  |
| Male | Ref | Ref | Ref | Ref |
| Female | 0.015^****^ | 0.024^****^ | 0.184^****^ | 0.208^****^ |
|  | 0.001 | 0.002 | 0.009 | 0.010 |
|  | 0.012,0.018 | 0.019,0.028 | 0.167,0.201 | 0.188,0.227 |
| **Payroll type** |  |  |  |  |
| Salary | Ref | Ref | Ref | Ref |
| Hourly | -0.008^****^ | -0.019^****^ | -0.077^****^ | -0.096^****^ |
|  | 0.002 | 0.004 | 0.016 | 0.017 |
|  | -0.012,-0.005 | -0.027,-0.011 | -0.108,-0.045 | -0.129,-0.062 |
| Unknown | -0.002 | -0.011^**^ | -0.003 | -0.013 |
|  | 0.002 | 0.005 | 0.023 | 0.024 |
|  | -0.007,0.002 | -0.020,-0.001 | -0.049,0.044 | -0.062,0.035 |
| **Type of work** |  |  |  |  |
| Intellectual work | Ref | Ref | Ref | Ref |
| Manual Work | -0.008^****^ | -0.016^****^ | 0.048^****^ | 0.032^**^ |
|  | 0.002 | 0.004 | 0.013 | 0.015 |
|  | -0.012,-0.004 | -0.023,-0.009 | 0.022,0.074 | 0.002,0.061 |
| Other | -0.003 | -0.004 | 0.077^**^ | 0.072^*^ |
|  | 0.003 | 0.005 | 0.038 | 0.038 |
|  | -0.009,0.003 | -0.014,0.005 | 0.001,0.152 | -0.005,0.149 |
| **Employer health plan indicator** |  |  |  |  |
| Self-funded plans | Ref | Ref | Ref | Ref |
| Fully insured health plans | -0.012^**^ | -0.018^***^ | -0.114^**^ | -0.132^***^ |
|  | 0.004 | 0.006 | 0.047 | 0.049 |
|  | -0.020,-0.003 | -0.031,-0.006 | -0.208,-0.019 | -0.229,-0.034 |
| **Rurality** |  |  |  |  |
| Rural | Ref | Ref | Ref | Ref |
| Urban | 0.011^****^ | 0.019^****^ | 0.032^*^ | 0.051^**^ |
|  | 0.002 | 0.005 | 0.019 | 0.022 |
|  | 0.006,0.015 | 0.009,0.029 | -0.006,0.071 | 0.008,0.095 |
| **Comorbidity Index** | 0.022^****^ | 0.038^****^ | 0.353^****^ | 0.391^****^ |
|  | 0.002 | 0.003 | 0.012 | 0.014 |
|  | 0.019,0.025 | 0.031,0.045 | 0.330,0.376 | 0.364,0.418 |
| **Covid cases**  (per 1,000,000 population) | 0.040^***^ | 0.057^**^ | -0.006 | 0.052 |
|  | 0.012 | 0.025 | 0.049 | 0.034 |
|  | 0.015,0.065 | 0.006,0.108 | -0.103,0.092 | -0.016,0.120 |
| **Constant** | 0.071^****^ | 0.134^****^ | 0.460^****^ | 0.594^****^ |
|  | 0.003 | 0.009 | 0.029 | 0.029 |
|  | 0.064,0.078 | 0.115,0.152 | 0.402,0.518 | 0.535,0.653 |
| Observations | 1,516,464 | 1,516,464 | 1,516,464 | 1,516,464 |

*p < 0.10 **p < 0.05 ***p < 0.01 ****p < 0.001

**Source:** Merative Commercial Claims and Encounters database 2019-2021

###### **Supplemental Table S2.12. DID Estimation: Outpatient Service Utilization among Individuals in Treated and Always Group**

|  | **Probability of**  **Any telehealth visits** | | **Number of**  **Telehealth visits** | **Number of**  **in-person visits** | **Number of**  **Total visits** |
| --- | --- | --- | --- | --- | --- |
|  | b/se/ci95 | b/se/ci95 | | b/se/ci95 | b/se/ci95 |
| **Treated** x **Post period** | 0.015^**^ | 0.034^**^ | | -0.023 | 0.012 |
|  | 0.007 | 0.016 | | 0.016 | 0.012 |
|  | 0.000,0.030 | 0.003,0.065 | | -0.054,0.009 | -0.013,0.036 |
| Age | -0.001^****^ | -0.002^****^ | | 0.001^****^ | -0.001 |
|  | 0.000 | 0.000 | | 0.000 | 0.000 |
|  | -0.001,-0.001 | -0.003,-0.002 | | 0.001,0.002 | -0.002,0.000 |
| **Sex** |  |  | |  |  |
| Male | Ref | Ref | | Ref | Ref |
| Female | 0.015^****^ | 0.024^****^ | | 0.184^****^ | 0.208^****^ |
|  | 0.001 | 0.002 | | 0.009 | 0.010 |
|  | 0.012,0.018 | 0.019,0.028 | | 0.167,0.201 | 0.188,0.227 |
| **Payroll type** |  |  | |  |  |
| Salary | Ref | Ref | | Ref | Ref |
| Hourly | -0.008^****^ | -0.019^****^ | | -0.076^****^ | -0.095^****^ |
|  | 0.002 | 0.004 | | 0.016 | 0.017 |
|  | -0.012,-0.005 | -0.027,-0.010 | | -0.108,-0.045 | -0.128,-0.062 |
| Unknown | -0.002 | -0.011^**^ | | -0.002 | -0.013 |
|  | 0.002 | 0.005 | | 0.023 | 0.024 |
|  | -0.007,0.002 | -0.020,-0.001 | | -0.049,0.044 | -0.062,0.035 |
| **Type of work** |  |  | |  |  |
| Intellectual work | Ref | Ref | | Ref | Ref |
| Manual Work | -0.008^****^ | -0.016^****^ | | 0.048^****^ | 0.032^**^ |
|  | 0.002 | 0.004 | | 0.013 | 0.015 |
|  | -0.012,-0.004 | -0.023,-0.009 | | 0.022,0.075 | 0.003,0.061 |
| Other | -0.003 | -0.004 | | 0.077^**^ | 0.073^*^ |
|  | 0.003 | 0.005 | | 0.037 | 0.038 |
|  | -0.009,0.003 | -0.014,0.005 | | 0.002,0.153 | -0.004,0.150 |
| **Employer health plan indicator** |  |  | |  |  |
| Self-funded plans | Ref | Ref | | Ref | Ref |
| Fully insured health plans | -0.012^**^ | -0.018^***^ | | -0.114^**^ | -0.132^***^ |
|  | 0.004 | 0.006 | | 0.047 | 0.048 |
|  | -0.020,-0.003 | -0.031,-0.006 | | -0.208,-0.019 | -0.229,-0.035 |
| **Rurality** |  |  | |  |  |
| Rural | Ref | Ref | | Ref | Ref |
| Urban | 0.011^****^ | 0.019^****^ | | 0.032^*^ | 0.051^**^ |
|  | 0.002 | 0.005 | | 0.019 | 0.021 |
|  | 0.006,0.015 | 0.009,0.029 | | -0.006,0.070 | 0.008,0.094 |
| **Comorbidity Index** | 0.022^****^ | 0.038^****^ | | 0.353^****^ | 0.391^****^ |
|  | 0.002 | 0.003 | | 0.012 | 0.014 |
|  | 0.019,0.025 | 0.031,0.045 | | 0.330,0.376 | 0.364,0.418 |
| **Covid cases**  (per 1,000,000 population) | 0.037^****^ | 0.048^**^ | | -0.002 | 0.046 |
|  | 0.009 | 0.019 | | 0.043 | 0.035 |
|  | 0.018,0.055 | 0.010,0.087 | | -0.088,0.084 | -0.024,0.117 |
| **Constant** | 0.073^****^ | 0.137^****^ | | 0.493^****^ | 0.630^****^ |
|  | 0.003 | 0.008 | | 0.028 | 0.029 |
|  | 0.066,0.079 | 0.120,0.154 | | 0.436,0.550 | 0.571,0.689 |
| R-squared | 0.052 | 0.040 | | 0.069 | 0.074 |

*p < 0.10 **p < 0.05 ***p < 0.01 ****p < 0.001

Source: Merative Commercial Claims and Encounters database 2019-2021

**Method**

We used a linear regression model with state and monthly fixed effects, specified as follows:

$$Y_{ist}= \beta_{0}+ \beta_{1} {TREAT}_{is}+{\beta_{2}*Postt+\beta}_{3}\left( {TREAT}_{is}\times Postt \right)+ \theta1X_{ist}+ {\theta_{2} X}_{st}+ \gamma_{s}+ \lambda_{t}+ \xi_{it}$$

where Y_ist_ is the outcome of interest (telehealth, in-person, and total outpatient service utilization). i represents individual, s represents state, and t represents month-year. $TREAT$_is_ is a binary variable equal to one if individual i lived in a treated state that ever adopted payment parity. *Post_t_* is a binary variable equal to one, if the time period is after March 2020, which indicates the post-period after adopting payment parity. X_ist_ is a vector of observed covariates representing individual-level demographic characteristics such as age, sex, occupation, comorbidity, rurality and health insurance type. X_st_ is a vector of time-varying state-level characteristics, including the COVID-19 cases in that state at time t. $\gamma$_s_ and λ_t_ represent a vector of the state and month and year-fixed effects, respectively, to account for unobserved state-level heterogeneity and assume any exogenous shock has an identical impact on each state. $\xi_{ist}$ is error term. The main coefficient of interest is 𝛽_3_$\left( {TREAT}_{is}\times Postt \right)$. 𝛽_3_represents the differences in the outcome variable between treatment and control states before and after payment parity. 𝛽_3_ is the causal effect of the policy change on health service utilization. Before analyzing the exogenous event specification, we tested the parallel trends assumption.

Note: When calculating the number of visits, we counted multiple claims on a single date for a single type of encounter as a single visit.
